# Income and Subjective Well-Being: New Insights from Relatively Healthy American Women, Ages 49-79

**DOI:** 10.1371/journal.pone.0146303

**Published:** 2016-02-01

**Authors:** Grace Wyshak

**Affiliations:** Harvard Medical School, Cambridge Hospital and Harvard T.H. Chan School of Public Health, Boston, Massachusetts, United States of America; University of Tuebingen Medical School, GERMANY

## Abstract

The interests of economists, psychologists, social scientists and others on the relations of income, demographics, religion and subjective well-being, have generated a vast global literature. It is apparent that biomedical research has focused on white with men. The Women’s Health Initiative and Observational Study (WHI OS) was initiated in 1992. The OS represents the scientific need for social priorities to improve the health and welfare of women; it includes 93.676 relatively healthy postmenopausal women, 49 to 79, from diverse backgrounds. The objective of this study is to examine how lifestyle and other factors influence women’s health. Data from the WHI OS questionnaire were analyzed. Statistical methods included descriptive statistics square, correlations, linear regression and analyses of covariance (GLM). New findings and insights relate primarily to general health, religion, club attendance, and likelihood of depression. The most important predictor of excellent or very good health is quality of life and general health is a major predictor of quality of life. A great deal of strength and comfort from religion was reported by 62.98% of the women, with little variation by denomination. More from religion related to poorer health, and less likelihood of depression. Religion and lower income are in accord with of across country studies. Attendance at clubs was associated with religion and with all factors associated with religion, except income. Though general health and likelihood of depression are highly correlated, better health is associated with higher income; however, likelihood of depression is not associated with income—contrary to conventional wisdom about socioeconomic disparities and mental health. Subjective well-being variables, with the exception of quality of life, were not associated with income. Social networks—religion and clubs—among a diverse population, warrant further attention from economists, psychologists, sociologists, and others.

## Introduction

Economists, sociologists, psychologists, epidemiologists and other researchers have generated a vast literature on the associations between sociodemographic and psychosocial factors, health, and subjective well-being. Some of the important factors that have been found to be associated with individual well-being include income; job, material, and life satisfaction; happiness; social networks and religion. However, these studies are characterized by great differences in methods, measurements, interpretation of empirical results and meta-analyses, as well as views on how and what to study. As a result, well-being has eluded definition [1], and overall “confusion abounds” in how best to measure life evaluation [2]. More research needs to focus on personal factors and life circumstances [3]. This point is illustrated by observations that the influence of religion on the determinants of economic growth has been neglected [4], and calls for research on religion as a social determinant of health [5]. (These citations and other relevant citations are in the S1 Appendix).

Furthermore, much of the earlier biomedical research on these questions had focused disproportionately on white men. Efforts to include women were made [6, 7]. In 1991 the WHI was launched by Dr. Bernadine Healy the first woman NIH Director.

In this study, I examined the associations among income, subjective well-being, likelihood of depression, health, happiness, religion/religiosity, club attendance, life evaluation, demographic and behavioral factors among healthy postmenopausal women. To do so, I used data from the 93,676 women, 49 to 79 years of age, who participated in the Women’s Health Initiative Observational Study (WHI OS), which uses well validated questions relating to income, subjective well-being, and other factors from widely used instruments, including the Medical Outcomes Study and the Rand 36-Item Health Survey (SF-36).

## Materials, Participants and Methods

During the initial phases of the WHI, the Institutional Review Boards at each of the 40 WHI clinical centers reviewed and approved the study. In the recent extension phases, the Fred Hutchinson Cancer Research Center has been the IRB of record for the WHI. Written informed consent is provided by participants (or next of kin) for review of medical records.

Women aged 49–79 who were ineligible or unwilling to participate in the clinical trial component of WHI or were recruited through a direct invitation for screening into the observational study. A total of 93,676 subjects were enrolled in the observational study. White women comprised 83% of the cohort (78,013), while 8% were Black (7,639) and 4% Hispanic (2,623). The remaining 5% of the cohort is comprised of Asian/Pacific Islander, American Indian, and subjects of unknown race/ethnicity. Religious denomination included 15 categories: 1) None (7.2%), 2) Catholic (27.1%), 3) Baptist (9.93%), 4) Episcopalian or Anglican (5.57%), 5) Lutheran (6.96%), 6) Methodist (10.28%), 7) Presbyterian (7.45%), 8) Adventist (0.42%), 9) Mormon/Latter Day Saints (0.78%), 10) Other Christian (10.17%), 11) Jewish (7.59%), 12) Eastern (Buddhist, Hindu) (0.75%), 13) Muslim (0.08%), 14) Other (4.88%), 15) Missing (0.82%).

### Materials

The data set analyzed is the WHI Baseline Data Set 10/16/2003, provided to me by the NHLBI (National Heart, Lung and Blood Institute).The Data Base Book consists of 1022 variables including demographics, eligibility for selection, personal information, medical history, reproductive history, family history, personal habits, thoughts and feeling, and other areas. It consists of well validated questions for determining subjective well-being, including those from widely used instruments including the Medical Outcomes Study, and the Rand 36-Item Health Survey (SF-36)[8] as well as constructs analogous to Cantril’s ladder [9]. The construction, reliability and validity of these instruments are considered in detail by others [10],

Measurements and definitions of variables may vary in the vast literature research conducted by economists, social scientists, psychologists, epidemiologists and others. Therefore, definitions in the WHI OS Data Set questionnaire for the 13 major variables, included in this study, are classified as follows: 1) Demographic, 2) Psychosocial/Behavioral, 3) Computed; and described in detail.

**Demographic**.

- Income “total family income (before taxes) from all sources within your household in the last year”. Income is coded in 10 categories: 1) less than $10,000 (4.18%), 2) $10,000–19,999 (10.79%), 3) $20,000–34,999 (21.60%), 4) $35,000–49,999 (18.61%), 5) $50,000–74,999 (18.67.2%), 6) $75,000–99,999 (8.74%), 7) $100, 000–149,999 (6.44%), 8) $150,000 or more (3.62%); and 9) “Don’t know” (2.98%) and 10) Missing (4.37%). The mode is in the $20,000–34,000 category, the median in the $35,000–49,999 category, interpolated median about $43,000 (excluding “Don’t know” and Missing).
- Education: 1) Didn’t go to school (.09%), 2) Grade school (1–4 years) (.38%), 3) Grade school (5–8 years) (1.20%) 4) Some high school (9–11 years) (3.51%), 5) High school diploma or GED (16.15%). 6) Vocational or Training School (9.74%), 7) Some college or Associate Degree (26.49%), 8) College graduate or Baccalaureate Degree (11.39%). 9) Some Postgraduate or professional (11.76%), 10) Master’s degree (15.73%), 11) Doctoral Degree (Ph.D., M.D., J.D., etc.) (2.76%), Missing (0.79%). The scale is condensed into 3 categories: 1) less than high school (22.12%). 2) high school to some college (47.63%) 3) college graduate or more (30.36%).
- Main job—present job or past job held the longest. Defined as “Managerial, professional specialty (Executive, managerial, administrative, professional occupations. Job titles include teacher, guidance counselor, registered nurse, doctor, lawyer, accountant, architect, computer/systems analyst, personnel manager, sales manager, etc.)—Missing, 4.7%”, No 54.02%, Yes 41.23%.

**Psychosocial/Behavioral**.

- Living alone”—Missing 0.8%, No 72.9%, Yes, 26.3%.
- General health—“In general, would you say your health is—on a five point scale: 1) Excellent’, 17.7%, 2) Very good, 40.2%, 3) Good, 31.7%, 4) Fair, 8.8%, 5) Poor, 0.9%, ‘Missing’ 0.7%.” For analyses the scale is coded: 0 = Missing 0.7%, 1 = Good/Fair/ Poor 41.37%, 2 = Excellent/Very/Good 57.93%.
- “Religion gives strength and comfort”—in three categories—None 12.51%, A little 24.01%, A great deal 62.98%, Missing, 0.50%. For analyses this is coded: 0 = Missing, 1 = None, 2 = A little, 3 = A great deal.
- “Attend clubs/lodges/groups.”—6 categories—1) not at all in the past month 43.9%; 2) once in the past month; 3) 2 or 3 times in the past month; 4) once a week 8.1%; 5} 2 or 6 times a week 5.6%; 6) every day 0.1%; missing 1.4%; For analyses the scale is coded: 0 = Missing, 1 = None 43.89%, 2 = Monthly 40.91%, 3 = Weekly or more13.84%.
- ‘Happy’: Questions are about how you feel and how things have been during the past 4 weeks. Give the one answer that comes closest to the way you have been feeling. Have you been happy?—on 6 point scale: All (10.13%), Most = (55.66), Good Bit = (15.69), Some = (11.93%), Little = (3.84%), None = 1 (1.06%.), Missing = 0 (0.07%)
- Satisfied with quality of life, analogous to Cantril’s ladder, 0-Dissatisfied to 10-Satisfied—Missing (0.76%), Dissatisfied (0.54%), 1 (0.39%), 2 (0.76%), 3 (1.49%), 4 (1.82%), Halfway (7.96%), 6 (4.14%), 7 (9.52%), 8 (22.44%), 9 (22.59%), Satisfied (27.59%). The scale is recoded—Missing = 0 (0.76%), Dissatisfied to 8 = 1 (49.06%), 9 to Satisfied = 2 (50.18%).
- Rate quality of life, also analogous to Cantril’s ladder, 0-Worst to 10-Best—Missing (0.77%), Worst (0.05%), 1 (0.06%), 2 (0.17%), 3 (0.44%), 4 (0.73%), Halfway (6.65%) 6 (3.35%), 7 (9.52%), 8 (29.82%), 9 (26.07%), Best (21.48%). The full scale is recoded—0 = missing (0.77%), Worst to 8 = 1 (51.68%), 9 to Best = 2 (47.55%).

**Computed**.

- “Likelihood of Depression” is a Shortened CES-D/DIS screening instrument; computed from Form 36/37, questions 100.1–100.6, 101, and 102. Source: Center for Epidemiological Studies; depression scale (CES-D, short form). PSHTDEP ranges from 0 to 1 with a higher score indicating a greater likelihood of depression. The distribution is highly skewed variable was dichotomized at greater than the median (0.00174). For analyses, the scale is coded: 0 = Missing (2.73%); 1 = Depressed (43.41%), range 0.00174 to 0.95938; 2 = Not Depressed, (53.87%), range 0.00028 to 0.00173.
- ‘Emotional well-being”, ranges from 0 to 100 with a higher score indicating a more favorable health state. The source of the scale is the Rand 36-Item Health Survey (SF-36). Computed from Form 36/37, questions 76, 77, 78, 80, and 82. Source: Rand 36-Item Health Survey (SF-36). For analyses, the scale is coded: 0 = Missing (1.57%), 1 = Least (22.86%) range 0 to 68, 2 = More (25.46%) range 72 to 80, 3 = Most emotional well-being (50.11%) range 84 to 100.
- “Social support’ is the sum of nine components. Scores range from 9 to 45, higher scores more support. The 9 components, each ranging from 1) None, 2) A little, 3) Some, 4) most, 5) All—of the time, are: Someone—a) ‘ to listen when need to talk’, b) ‘ to give good advice’; c) ‘who can take you to the doctor’, d) ‘to have a good time with’, e) ‘to help understand a problem when you need it’, f) ‘to help with daily chores if you are sick’, g) ‘to share your private worries’, h) ‘to do something fun with’, i) ‘to love you and make you feel wanted’. For analyses the scale for Social Support is coded: 0 = Missing (2.77%), 1 = Some (50.21%) range 9–37, 2 = Most (47.01%) range 38–45.
- Abbreviations for the 13 variables described above are used in text and tables. They are: Income, Education, Job, Live Alone, Health, Religion, Clubs, Satisfied w/life, Quality of Life, Depressed, Well-being, Happy, and Social Support.

#### Statistical Methods

Methods included descriptive statistics (means, standard deviations, etc.), chi-square analyses for categorical data, correlations, Multivariate linear regression and MANOVA (Multivariate analyses of covariance). SAS 9.3 software [11] was used.

## Results

Descriptive data for the 13 variables of primary interest in this study are mean values (Table 1) and frequency distributions (Table 2). Differences between Non-White and White women were negligible; White Women were older—63.9 compared to 62.2; had higher incomes, on a scale of 1 to 8, means are 4.16 vs. 4.12; and better ratings of general health, on a scale to 1 to 5,means are 2.34 vs. 2.37. Notably, general health and the subjective well-being ratings were high—the modes (most frequent responses) were: general health, 2 (very good); happiness, 5; emotional well-being, 88; satisfaction with life, 10; quality of life, 8; social support, 45; and rating); and modes of Non-white and White women differed slightly. Income–denoted in 8 categories—1. < $10,000 to 8. -<$150,000 or more–is normally distributed.

**Table 1:** Continuous Variable Descriptive Data.

Variable	N	Mean	Std Dev	Median	Mode	Min	Max
**Age**	93676	63.619	7.370	64	67	49	79
**Income 1–8^**	86792	4.154	1.706	4	3	1	8
**Education 1–3^^**	93676	2.081	0.719	2	2	1	3
**Managerial/Professional Job%**	89230	0.433	0.495	0	0	0	1
General Health 1–5*	93022	2.346	0.903	2	2	1	5
Likelihood of Depression 0–100*	91123	0.0420	0.132	0.00173	0.00144	0.00028	0.95938
**Happy 1–6^^^**	93019	4.545	1.026	5	5	1	6
**Emotion Well-being 0–100**	92209	78.569	14.710	84	88	0	100
**Satisfied 0–10 Dissat-Sat**	92965	8.098	1.950	9	**10**	0	**10**
**Quality of Life 0–10 Worst-Best**	92953	8.252	1.503	8	8	0	10
**Social Support 9–45 Low to High**	91079	35.917	7.858	37	**45**	9	**45**
**Nine Components of Social Support**							
**Listen 1–5**	93164	4.069	1.012	4	5	0	5
**Good Advice 1–5**	92978	3.837	1.099	4	4	0	5
**Take to Doctor 1–5**	92848	4.231	1.165	5	5	0	5
**Good Time 1–5**	92795	4.015	1.037	4	5	0	5
**Help w/Problems 1–5**	92887	3.923	1.079	4	4	0	5
**Help w/Chores 1–5**	92931	3.668	1.355	4	5	0	5
**Share 1–5**	92885	3.764	1.285	4	5	0	5
**Have Fun 1–5**	92955	3.959	1.049	4	5	0	5
**Love/Feel wanted 1–5**	92826	4.095	1.188	5	5	0	5

% Percent Yes.

^ 1) <$10k-, 2) $10k-, 3) $20k-, 4) $35k-, 5) $50k-, 6) $75k-, 7) $100k-, 8) > $150k. Excludes Missing and Don't Know.

^^ 1)High School of less; 2) Vocational, Some College, College Grad, 3) Post Grad, Masters, Doctoral Degree.

^^^ 1) None of the time, 2) A little bit, 3) Some, 4) A good bit, 5) Most, 6) All of the time.

* Low vales—better outcome.

1 Excellent, 2 Very Good, 3 Good, 4 Fair, 5 Poor.

N.B. Results exclude missing data.

**Table 2:** Percent Distributions of Categorical and Categorized Variables.

Income		Education—0 to 11	%	Managerial/Professional Job	%
	%	**0. 0**	0.79	**Missing**	4.75
**Less than $10,000**	4.51	**1. Didn't go to school**	0.09	**No**	54.02
**$10,000 to $19,999**	11.64	**2. Grade school (1–4 years)**	0.38	**Yes**	41.23
**$20,000 to $34,999**	23.31	**3. Grade school (5–8 years)**	1.20		
**$35,000 to $49,999**	20.09	**4. Some high school (9–11 years)**	3.51		
**$50,000 to $74,999**	20.15	**5. High school diploma or GED**	16.15	General Health*	%
**$75,000 to $99,999**	9.43	**6. Vocational or training school**	9.74	**Missing**	0.70
**$100,000 to $149,999**	6.95	**7. Some college or Associate Degree**	26.49	**Good/fair/poor**	41.38
**$150,000 or more**	3.91	**8. College graduate or Baccalaureate Degree**	11.39	**Excellent/Very Good**	57.92
		**9. Some post-graduate or professional**	11.76		
**Ethnic/Race**	%	**10. Master's Degree**	15.73	Likelihood of Depression*	%
**MISSING**	0.28	**11. Doctoral Degree (Ph.D,M.D.,J.D.,etc.)**	2.76	**Missing**	2.73
**American Indian or Alaskan Native**	0.45			**Yes (.0074-.95938)**	43.41
**Asian or Pacific Islander**	2.85	**Education 3 Categories**	%	**No (.00038-.0073)**	53.87
**Black or African-American**	8.15	**1. 0 to 6–0 to High School Diploma or GED**	22.12		
**Hispanic/Latino**	3.87	**2. 6 to 8—Vocational to College Grad**	47.63	**Happiness**	%
**White (not of Hispanic origin)**	83.28	**3. 9 to 11—Post Grad to Doctoral Degree**	30.36	**Missing**	0.70
**Other**	1.11			**None**	1.06
		**Attend Club/Lodges^**	%	**A little bit**	3.84
**Religion**	**%**	**^How often have you gone to**		**Some**	11.93
**None**	7.2	**meetings of clubs, lodges,etc.?**		**A good bit**	15.69
**Catholic**	27.1	**1. MISSING**	1.36	**Most**	56.66
**Baptist**	9.93	**2. Not at all in the past month**	43.89	**All of the time**	10.13
**Episcopalian or Anglican**	5.57	**3. Once in the past month**	18.46		
**Lutheran**	6.96	**4. 2 or 3 times in the past month**	22.45	**Emotional Well-being**	%
**Methodist**	10.28	**5. Once a week**	8.08	**Missing**	1.57
**Presbyterian**	7.45	**6. 2 or 6 times a week**	5.65	**Little (0–68)**	22.86
**Adventist**	0.42	**7. Every day**	0.10	**Some (72–80)**	25.46
**Mormon/Latter Day Saints**	0.78			**Most (84–100)**	50.11
**Other Christian**	10.17	**Club Attendance 4 categories**	%		
**Jewish**	7.59	**1. Missing**	1.36	Satisfaction with Life*	%
**Eastern (Buddhist, Hindu)**	0.75	**2. None**	43.89	**Missing**	0.76
**Muslim**	0.08	**3. Monthly**	40.91	**Less (Dissat-8)**	49.06
**Other Christian**	4.88	**4. Weekly or more**	13.84	**More (9-Satified)**	50.18
**Missing**	0.82				
		**Strength, Comfort from Religion**	%	Quality of Life*	%
		**Missing**	0.50	**Missing**	0.77
		**None**	12.51	**Lower Rating (Worst-8)**	51.68
		**A Little**	24.01	**Higher Rating (9-Best)**	47.55
		**A Great Deal**	62.98		
				Social Support*	%
				**Missing**	2.77
				**Less (9–37)**	50.21
				**More (38–45)**	47.01

*Categorized at or about the median.

N.B Parentheses show ranges.

Results of frequency distributions for categorical variables: income, education, ‘strength and comfort from religion’, having/had a professional managerial job, ‘attend clubs/lodges/groups’, living alone (no/yes) and the following subjective well-being variables that have been categorized: general health, happiness, satisfaction with life, rating of quality of life, social support, emotional well-being, and likelihood of depression. Income conforms closely to a normal distribution—40% reported incomes between $35,000 and less than $75,000. Fewer the 25% were in the range of no education to a high school had diploma or GED, 30% had post-graduate to doctoral degrees. Variables with asterisks (*) were dichotomized at or close to the median. (Missing values, in the 10 of the 13 are about 1%; exceptions are professional/managerial job, 4.7%, and two computed variables—likelihood of depression, 2.7%, and social support, 2.8%.)

Results of multivariate linear regression forward selection analyses for 13 variables (income, education, religion, clubs job, live alone, health, depression, happy, well-being, satisfied with life, quality of life, and social support); each as a dependent and an independent variable, displayed by clusters (Table 3); and results of correlations (Table 4). Supporting data are in S1 Table Linear Regression results that include parameter estimates and R-Sqs. and S2 Table MANOVA results.

**Table 3:** Multivariate Linear Regression Forward Selection.

**Income**							**Education**						
**Summary of Forward Selection**							**Summary of Forward Selection**						
**Step**	**Variable**	**Partial**	**Model**	**C(p)**	**F Value**	**Pr > F**	**Step**	**Variable**	**Partial**	**Model**	**C(p)**	**F Value**	**Pr > F**
	**Entered**	**R-Square**	**R-Square**					**Entered**	**R-Square**	**R-Square**			
**1**	**Education**	0.1252	0.1252	1883.63	**12424.10**	< .0001	**1**	**Job**	0.2224	0.2224	6462.38	**24826.80**	< .0001
**2**	**Job**	0.0159	0.1411	272.25	**1608.40**	< .0001	**2**	**Income**	0.0538	0.2762	18.34	**6444.90**	< .0001
**3**	**Live Alone**	0.0018	0.1430	88.51	185.56	< .0001	**3**	**Live Alone**	0.0002	**0.2763**	1.86	18.48	< .0001
**4**	**Health**	0.0004	0.1434	48.18	42.31	< .0001	**NS**	**Social Support**	**Quality of Life**	**Satisfied w/Life**	**Depressed**	**Health**	**Clubs**
**5**	**Religion**	0.0002	0.1436	30.21	19.97	< .0001	**P-values**	0.8425	0.6316	0.6913	0.5924	0.5316	0.5242
**6**	**Quality of Life**	0.0002	**0.1438**	7.35	24.86	< .0001	**NS**	**Happy**	**Well-being**	**Religion**			
**NS**	**Depressed**	**Happy**	**Satisfied w/Life**	**Clubs**	**Well-being**	**Social Support**	**P-values**	0.3582	0.1369	0.1301			
**P-values**	0.9961	0.8577	0.5952	0.2633	0.1317	0.1130							
**Religion Gives Strength and Comfort**							**Attend clubs/lodges/groups**						
**Summary of Forward Selection**							**Summary of Forward Selection**						
**Step**	**Variable**	**Partial**	**Model**	**C(p)**	**F Value**	**Pr > F**	**Step**	**Variable**	**Partial**	**Model**	**C(p)**	**F Value**	**Pr > F**
	**Entered**	**R-Square**	**R-Square**					**Entered**	**R-Square**	**R-Square**			
**1**	**Happy**	0.0137	0.0137	2559.35	**1201.93**	< .0001	**1**	**Religion**	0.0132	0.0132	1512.08	1161.83	< .0001
**2**	**Clubs**	0.0106	0.0242	1605.23	**938.79**	< .0001	**2**	**Happy**	0.0094	0.0226	674.01	833.62	< .0001
**3**	**Health**	0.0075	0.0317	928.02	672.06	< .0001	**3**	**Live Alone**	0.0037	0.0263	346.02	328.7	< .0001
**4**	**Satisfied w/Life**	0.0070	0.0387	294.60	633.31	< .0001	**4**	**Quality of life**	0.0019	0.0282	175.46	172.23	< .0001
**5**	**Social Support**	0.0012	0.0399	187.21	109.16	< .0001	**5**	**Depressed**	0.0009	0.0292	93.50	83.87	< .0001
**6**	**Quality of Life**	0.0006	0.0405	136.14	52.99	< .0001	**6**	**Health**	0.0006	0.0297	43.64	51.84	< .0001
**7**	**Well-being**	0.0006	0.0411	87.38	50.72	< .0001	**7**	**Job**	0.0001	0.0299	32.61	13.03	0.0003
**8**	**Income**	0.0005	0.0416	39.86	49.5	< .0001	**8**	**Satisfied w/Life**	0.0001	0.0300	22.02	12.59	0.0004
**9**	**Depressed**	0.0001	0.0418	28.37	13.49	0.0002	**9**	**Well-being**	0.0001	0.0301	16.35	7.67	0.0056
**10**	**Job**	0.0001	0.0419	20.04	10.33	0.0013	**10**	**Social Support**	0.0001	**0.0302**	11.84	6.51	0.0107
**11**	**Live Alone**	0.0001	**0.0420**	12.00	10.04	0.0015	**NS**	**Education**	**Income**				
**NS**	**Education**						**P-values**	0.5002	0.1747				
**P-value**	0.1096												
**Managerial/Professional Job No/Yes**							**Live Alone No/Yes**						
**Summary of Forward Selection**							**Summary of Forward Selection**						
**Step**	**Variable**	**Partial**	**Model**	**C(p)**	**F Value**	**Pr>F**	**Step**	**Variable**	**Partial**	**Model**	**C(p)**	**F Value**	**Pr > F**
	**Entered**	**R-Square**	**R-Square**					**Entered**	**R-Square**	**R-Square**			
**1**	**Education**	0.2224	0.2224	1626.02	24826.80		**1**	**Social Support**	0.0349	0.0349	1023.92	**3136.83**	< .0001
**2**	**Income**	0.0141	0.2366	19.32	1608.40		**2**	**Clubs**	0.0045	0.0393	619.62	**403.43**	< .0001
**3**	**Live Alone**	0.0001	0.2366	14.62	6.70		**3**	**Well-being**	0.0016	0.0409	478.54	142.31	< .0001
**4**	**Clubs**	0.0001	0.2367	10.84	5.78		**4**	**Quality of Life**	0.0027	0.0437	231.91	247.98	< .0001
**5**	**Religion**	0.0001	0.2367	6.80	6.03		**5**	**Income**	0.0013	0.045	113.30	120.47	< .0001
**6**	**Health**	0.0000	0.2368	4.67	4.14		**6**	**Health**	0.0005	0.0455	68.71	46.55	< .0001
**NS**	**Well-being**	**Depressed**	**Quality of Life**	**Social Support**	**Happy**	**Satisfied w/Life**	**7**	**Education**	0.0004	0.0458	38.85	31.85	< .0001
**P-values**	0.6072	0.6456	0.5678	0.5183	0.2262	0.3214	**8**	**Satisfied w/Life**	0.0002	0.0460	24.43	16.42	< .0001
							**9**	**Religion**	0.0001	0.0461	15.84	10.59	0.0011
							**10**	**Job**	0.0001	**0.0462**	12.13	5.71	0.0169
							**NS**	**Happy**	**Depressed**				
							**P-values**	0.4272	0.1138				
**Health**							**Depressed**						
**Summary of Forward Selection**							**Summary of Forward Selection**						
**Step**	**Variable**	**Partial**	**Model**	**C(p)**	**F Value**	**Pr > F**	**Step**	**Variable**	**Partial**	**Model**	**C(p)**	**F Value**	**Pr > F**
	**Entered**	**R-Square**	**R-Square**					**Entered**	**R-Square**	**R-Square**			
**1**	**Quality of Life**	0.0945	0.0945	5665.11	9055.07	< .0001	**1**	**Well-being**	0.2030	0.203	2068.7	22099.7	< .0001
**2**	**Well-being**	0.0398	0.1343	1604.23	3989.27	< .0001	**2**	**Satisfied w/Life**	0.0109	0.2138	858.068	1200.8	< .0001
**3**	**Religion**	0.0072	0.1415	871.96	727	< .0001	**3**	**Social Support**	0.0030	0.2169	521.55	336.51	< .0001
**4**	**Depressed**	0.0033	0.1448	535.449	336.45	< .0001	**4**	**Health**	0.0027	0.2196	217.864	304.94	< .0001
**5**	**Satisfied w/Life**	0.0026	0.1474	272.318	264.32	< .0001	**5**	**Happy**	0.0012	0.2209	81.2651	138.48	< .0001
**6**	**Social Support**	0.0012	0.1485	154.762	119.35	< .0001	**6**	**Clubs**	0.0005	0.2214	21.9513	61.3	< .0001
**7**	**Clubs**	0.0006	0.1492	94.3274	62.37	< .0001	**7**	**Quality of Life**	0.0001	0.2215	10.082	13.87	0.0002
**8**	**Live Alone**	0.0005	0.1497	44.6643	51.64	< .0001	**8**	**Religion**	0.0000	0.2216	6.9737	5.11	0.0238
**9**	**Income**	0.0003	0.1499	19.8091	26.85	< .0001							
**10**	**Happy**	0.0001	0.1500	13.0307	8.78	0.0030	**NS**	**Education**	**Live Alone**	**Job**	**Income**		
**11**	**Job**	0.00000	0.1500	11.3832	3.65	0.0562	**P-Values**	0.3345	0.3868	0.5917	1.00E+00		
**NS**	**Job**												
**P-Values**	0.0562												
**Happy**							**Emotional Well-being**						
**Summary of Forward Selection**							**Summary of Forward Selection**						
**Step**	**Variable**	**Partial**	**Model**	**C(p)**	**F Value**	**Pr > F**	**Step**	**Variable**	**Partial**	**Model**	**C(p)**	**F Value**	**Pr > F**
	**Entered**	**R-Square**	**R-Square**				**1**	**Entered**	**R-Square**	**R-Square**			
**1**	**Well-being**	0.4921	0.4921	8168.82	**84082.2**	< .0001	**2**	**Happy**	0.4921	0.4921	9798.74	**84082.2**	< .0001
**2**	**Satisfied w/Life**	0.0278	0.5199	2971.21	**5027.67**	< .0001	**3**	**Depressed**	0.0387	0.5308	2432.50	**7167.60**	< .0001
**3**	**Social Support**	0.0058	0.5257	1889.07	**1061.09**	< .0001	**4**	**Satisfied w/Life**	0.0068	0.5376	1146.89	**1270.88**	< .0001
**4**	**Depressed**	0.0026	0.5283	1399.15	484.15	< .0001	**5**	**Health**	0.0042	0.5418	345.35	800.4	< .0001
**5**	**Religion**	0.0026	0.5309	911.32	484.77	< .0001	**6**	**Social Support**	0.0007	0.5425	210.30	136.72	< .0001
**6**	**Health**	0.0028	0.5338	387.06	523.96	< .0001	**7**	**Live Alone**	0.0007	0.5432	82.18	130.01	< .0001
**7**	**Quality of Life**	0.0014	0.5352	122.34	266.37	< .0001	**8**	**Quality of Life**	0.0002	0.5434	47.44	36.73	< .0001
**8**	**Clubs**	0.0006	**0.5358**	6.90	117.44	< .0001	**9**	**Religion**	0.0002	0.5436	15.13	34.30	< .0001
**NS**	**Income**	**Job**	**Education**	**Live Alone**			**10**	**Clubs**	0.0000	0.5436	10.01	7.13	0.0076
**P-values**	0.8578	0.4968	0.5573	0.4230			**11**	**Education**	0.0000	**0.5437**	10.18	4.23	0.0397
							**NS**						
							**P-values**	**Job**	**Income**				
								0.6072	0.3379				
**Satisfaction with Life**							**Quality of Life**						
**Summary of Forward Selection**							**Summary of Forward Selection**						
**Step**	**Variable**	**Partial**	**Model**	**C(p)**	**F Value**	**Pr > F**	**Step**	**Variable**	**Partial**	**Model**	**C(p)**	**F Value**	**Pr > F**
	**Entered**	**R-Square**	**R-Square**					**Entered**	**R-Square**	**R-Square**			
**1**	**Quality of Life**	0.5921	0.5921	5653.08	**126003.00**	< .0001	**1**	**Satisfied w/Life**	0.5921	0.5921	3722.43	126003.00	< .0001
**2**	**Happy**	0.0169	0.6091	1820.72	**3755.7**	< .0001	**2**	**Health**	0.0074	0.5996	2071.48	1614.47	< .0001
**3**	**Social Support**	0.0039	0.6129	943.47	**869.84**	< .0001	**3**	**Social Support**	0.0050	0.6046	968.35	1092.98	< .0001
**4**	**Well-being**	0.002	0.6149	502.25	440.7	< .0001	**4**	**Happy**	0.0033	0.6079	233.49	734.93	< .0001
**5**	**Depressed**	0.0008	0.6157	314.89	188.68	< .0001	**5**	**Live Alone**	0.0003	0.6082	161.06	74.3	< .0001
**6**	**Health**	0.0006	0.6164	171.98	144.64	< .0001	**6**	**Religion**	0.0003	0.6085	100.75	62.24	< .0001
**7**	**Religion**	0.0006	0.6170	31.56	142.38	< .0001	**7**	**Well-being**	0.0002	0.6087	50.03	52.69	< .0001
**8**	**Live Alone**	0.0001	0.6171	17.94	15.62	< .0001	**8**	**Clubs**	0.0001	0.6088	31.26	20.77	< .0001
**9**	**Clubs**	0.0000	**0.6171**	10.11	9.82	0.0017	**9**	**Income**	0.0001	0.6089	17.82	15.44	< .0001
**NS**	**Income**	**Education**	**Job**				**NS**		**Education**	**Job**			
**P-values**	0.6082	0.4560	0.1931				**P-values**		0.6448	0.6737			
**Social Support**													
**Summary of Forward Selection**													
**Step**	**Variable**	**Partial**	**Model**	**C(p)**	**F Value**	**Pr > F**							
	**Entered**	**R-Square**	**R-Square**										
**1**	**Satisfied w/Life**	0.1143	0.1143	7625.81	11203.1	< .0001							
**2**	**Happy**	0.0317	0.1461	4245.45	3224.72	< .0001							
**3**	**Live Alone**	0.0255	0.1715	1531.85	2668.63	< .0001							
**4**	**Quality of Life**	0.0071	0.1787	774.45	752.73	< .0001							
**5**	**Depressed**	0.0035	0.1821	406.14	368.60	< .0001							
**6**	**Well-being**	0.0018	0.1839	216.94	190.74	< .0001							
**7**	**Religion**	0.0009	0.1849	117.85	100.96	< .0001							
**8**	**Health**	0.0009	0.1858	18.61	101.23	< .0001							
**9**	**Clubs**	0.0001	0.1859	14.01	6.60	0.0102							
**10**	**Income**	0.0000	**0.1859**	10.73	5.28	0.0216							
**NS**	**Education**	**Job**											
**P-values**	0.8425	0.3918											

**Table 4:** Pearson Correlation Coefficients.

	Income	Education	Clubs	Religion	Health	Depressed	Live alone	Job	Happy	Well-Being	Satislfied w Life	Quality of Life	Social Support
**Income**	1	**0.35387**	0.00548	**-0.02186**	**0.02638**	0.00787	**-0.04098**	**0.27814**	0.01187	0.00897	**0.01915**	**0.02614**	**0.02174**
		***	**0.1062**	***	***	0.0204	***	***	0.0005	0.0082	***	***	***
**Education**	**0.35387**	1	0.00977	**-0.01561**	0.01231	0.00343	0.00204	**0.47597**	0.0079	0.01263	0.0046	0.00881	0.00641
	***		0.0028	***	0.0002	**0.2940**	0.5333	***	0.0156	0.0001	**0.1590**	0.0070	0.0496
**Clubs**	0.00548	0.00977	1	0.11541	0.05787	0.07239	0.05575	0.0113	0.06018	0.07786	0.08651	0.08516	0.04951
	**0.1062**	0.0028		***	***	***	***	0.0005	***	***	***	***	***
**Religion**	**-0.02186**	**-0.01561**	**0.11541**	1	**-0.04635**	**0.03252**	-0.00337	**-0.01551**	**0.06532**	**0.06108**	**0.11337**	**0.10503**	**0.08016**
	***	***	***		***	***	**0.3027**	***	***	***	***	***	***
**Health**	**0.02638**	0.01231	**0.05787**	**-0.04635**	1	**0.20633**	-0.00605	**0.01492**	**0.2241**	**0.27388**	**0.29254**	**0.30772**	**0.17036**
	***	0.0002	***	***		***	**0.0639**	***	***	***	***	***	***
**Depression**	0.00787	0.00343	**0.07239**	**0.03252**	**0.20633**	1	**-0.0242**	0.00307	**0.32134**	**0.40064**	**0.27537**	**0.25346**	**0.20192**
	0.0204	**0.2940**	***	***	***		***	**0.3477**	***	***	***	***	***
**Live alone**	**-0.04098**	0.00204	**0.05575**	-0.00337	-0.00605	**-0.0242**	1	0.00294	**-0.07661**	0.00314	**-0.08566**	**-0.09195**	**-0.18541**
	***	**0.5333**	***	**0.3027**	**0.0639**	***		**0.3679**	***	**0.3363**	***	***	***
**Job**	**0.27814**	**0.47597**	0.0113	**-0.01551**	**0.01492**	0.00307	0.00294	1	0.00667	0.01214	0.00173	0.00472	0.00558
	***	***	0.0005	***	***	**0.3477**	**0.3679**		0.0411	0.0002	**0.5961**	**0.1482**	**0.0876**
**Happy**	0.01187	0.0079	**0.06018**	**0.06532**	**0.2241**	**0.32134**	**-0.07661**	0.00667	1	**0.49837**	**0.37741**	**0.35233**	**0.26415**
	0.0005	0.0156	***	***	***	***	***	0.0411		***	***	***	***
** Well-being**	0.00897	0.01263	**0.07786**	**0.06108**	**0.27388**	**0.40064**	0.00314	0.01214	**0.49837**	1	**0.3707**	**0.34817**	**0.25724**
	0.0082	0.0001	***	***	***	***	**0.3363**	0.0002	***		***	***	***
**Satisfied/Life**	**0.01915**	0.0046	**0.08651**	**0.11337**	**0.29254**	**0.27537**	**-0.08566**	0.00173	**0.37741**	**0.3707**	1	**0.76965**	**0.33757**
	***	**0.1590**	***	***	***	***	***	**0.5961**	***	***		***	***
**Quality of Life**	**0.02614**	0.00881	**0.08516**	**0.10503**	**0.30772**	**0.25346**	**-0.09195**	0.00472	**0.35233**	**0.34817**	**0.76965**	1	**0.33274**
	***	0.0070	***	***	***	***	***	**0.1482**	***	***	***		***
**Social Support**	**0.02174**	0.00641	**0.04951**	**0.08016**	**0.17036**	**0.20192**	**-0.18541**	0.00558	**0.26415**	**0.25724**	**0.33757**	**0.33274**	1
	***	0.0496	***	***	***	***	***	0.0876	***	***	***	***	

N.B. Abbreviations for the 13 variables described above are used in text and tables—Income, Education, Job, Live Alone, Health, Religion, Clubs, Satisfied w/life, Quality of Life, Depressed, Well-being, Happy, Social Support.

Correlation Coefficient in row of variable.

*** P<0.0001. Bold and Underlined—Not Significant. Else P-values Less than 0.0001.

### Cluster 1: Income, Education, Managerial/Professional Job

#### Income

For income as the dependent variable, significantly associated variables are: 6 independent variables: more education, managerial job, not living alone, better general health, less from religion, and better quality of life—the only significant subjective well-being variable—for all 6 P <0.0001. Not significantly associated with income are 4 subject well-being variables: happiness; satisfaction with life, emotional well-being and social support; and attendance at clubs and likelihood of depression. For income as the independent variable, 7 dependent variables are statistically significant: education, job, religion, general health, not living alone, quality of life, 6 with P <0.0001, the 7th social support,—P = 0.0216, Partial R-Sq. = 0.0000. Not significant associations are: club attendance and likelihood of depression.

Note: Significant associations are bidirectional except for social support that was not associated income as the dependent variable, P = 0.1130. But income as the independent variable and social support as the dependent variable are marginally associated, P = 0.0216 (Table 3).

#### Education

For education as the dependent variable, 3 variables are significantly associated—higher income, have managerial/professional job, and not living alone (Table 3). For education as the independent variable, higher income, job and not living alone are statistically significant. In addition, emotional well-being is marginally significant—Partial R-Sq. = 0.000, P = 0.0397 (Table 3).

#### Managerial/Professional Yes/No

For job as the dependent variable, significantly associated variables are: income, P<0.0001, education, P<0.0001, not living alone, P = 0.0096, more club attendance, P = 0.0162, less religion, P = 0.0140, better general health, P = 0.0420. The 5 subjective well-being variables and likelihood of depression are not (Table 3). For job as the independent variable, significantly associated variables are: higher income, P<0.0001, more education, P<0.0001, not living alone, P = 0.0169, more club attendance, P = 0.0003 and less religion, P = 0.0013; 7 variables not significant are: general health, likelihood of depression, and the 5 subjective well-being variables are (Table 3).

New findings: The correlations of income with education, job, religion, general health, living alone or not, satisfaction with life, quality of life and social support are significant (P<0.0001). For the remaining 4 variables correlations were smaller: with club attendance P = 0.3098, with likelihood of depression P = 0.0336, with happiness P = 0.0005, and with emotional well-being P = 0.0006 (Table 3). Multivariate linear regression results mitigate considerably the univariate associations. Income, education and job are not significantly associated with subjective well-being variables with one exception. Income and quality of life are associated bidirectionally (Table 3).

### Cluster 2: Two Social Networks—Strength and Comfort from Religion and Attendance at Clubs/Lodges, etc.

Social Networks here defined as religion/religiosity and club attendance, two strongly related variables, r = 0.10154, P<0.0001(Table 3). Both are associated with subjective well-being, and virtually all demographic and behavioral variables. Table 3. For religion as the dependent variable significant associations are with 11 of 12 independent variables are significant with P<0.0001; smaller P values are for: likelihood depression, P = 0.0002, job, P = 0.0013, and live alone, P = 0.0015. Education is the only variable not significantly associated with religion. Table 3. For religion as the independent variable, significant associations are with 11 of 12 dependent variables. P-values are <0.0001 for income, clubs, general health and the 5 subjective well-being variables. P = 0.0011 for live alone/or not and P = 0.0140 for job (Table 3).

For clubs as the dependent variable significant associations are with 10 of 12 independent variables, with exceptions income and education, and P values <0.0001 for 6 variables. Lower P-values are with job, P = 0.0003, satisfaction with life, P = 0.0004, emotional well-being, P = 0.0056, and social support, P = 0.0107 significant associations are with 10 of 12 independent variables, with exceptions income and education (Table 3). For clubs as the independent variable significant associations are with 10 of 12 independent variables, with exceptions income and education. P-values are modified slightly compared to P-values for religion as a dependent variable: job, P = 0.0162, emotional well-being P = 0.0076, satisfaction with life, P = 0.0017, and social support, P = 0.0102 (Table 3).

One important difference between clubs and religion is the finding that income and religion are associated—more religion and lower income (with each as the dependent and independent variable); correlation r = -0.0219, P<0.0001 (Table 4). In contrast, club attendance was not associated with income (with each as the dependent and independent variable) and correlations: r = 0.0055, P = 0.1062. A second difference relates to general health; for religion, poorer health more religion; for clubs poorer health less club attendance (Table 3).

### Cluster 3: Two Health Conditions—General health—Good/Fair/Poor Compared with Excellent/Very Good and Likelihood of Depression—Depressed Compared with Not Depressed

For general health and likelihood of depression r = 0.20633 (Table 4). However, as dependent and independent variables, the results of Multivariate linear regression show significant differences General health as the dependent variable is associated with all independent variables, except education and job. Likelihood of depression is as the dependent variable is associated with all independent variables except income, education, job, and live alone (Table 3). General health as the independent variable is associated with all dependent variables except education and job. However, likelihood of depression as the independent variable is not associated with income, education, job, and live alone—similar to results as the dependent variable; it is associated with all other variables (Table 3).

A notable result: quality of life is more highly correlated with general health than any other variable—r = 0.3772, P<0.0001 (Table 4). Also, with general health as the dependent variable, quality of life is primary independent variable; income is last out of 10. The differences for likelihood of depression are: quality of life ranks last of 8; emotional well-being is primary; followed by satisfaction with life and happiness then general health ranking 4^th^ out of 8 (Table 3). General health as the independent variable is a significant predictor for 11 dependent variables, 10 with P<0.0001; for job P = 0.0420, but not for education. Likelihood of depression as the independent variable is significant predictor for religion, P<0.0001, clubs, P<0.0001, 4 variables—happiness, emotional well-being, satisfaction with life and social support, P<0.0001—and quality of life, P = 0.0019; it is not significant predictor of income, education, job, and live alone or not (Table 3).

(Note: general health is on a five point scale of poor to excellent. “Likelihood of Depression” is from CES-D short form; it ranges from 0–100. It is dichotomized close to the median—depressed/not depressed.)

### Cluster 4: Subjective Well-Being—1) Happiness, 2) Emotional Well-Being, 3) Satisfaction with Life, 4) Quality of Life and 5) Social Support

Not surprisingly, these 5 variables are highly correlated (Table 4) However, there are notable differences—satisfaction with life and quality of life are 2 highly correlated variables—r = 0.76975, P <0.0001 (Table 4). However, results of Multivariate linear regression—forward selection—show differences in associations with other variables (Table 3).

The 5 dependent variables are significantly associated with religion, clubs, general health, and likelihood of depression. Not significant associations are:1) happiness with income, education, job and live alone; 2) emotional well-being with job and income; 3) satisfaction with life and income, education and job; 4) quality of life with education and job; 5) social support with education and job. Table 3. Among the 5 as independent variables, 3 are noteworthy—1) quality of life and income are significantly associated; it ranks 6^th^ among 6, Partial R-Sq. = 0.0002, P<0.0001; 2) social support and income are significantly associated. Also noteworthy, 3) happiness is not associated with live alone/or not. With few exceptions subjective well-being is not associated with economic factors—income, education and job, but it is important for health, religion and clubs (Table 3).

### Cluster 5: Live Alone or Not Alone

For live alone or not as the dependent variable significant associations are: 10 out 12 variables income to social support—primary variable Partial R-Sq. = 0.0349, 75% of Model R-Sq. = 0.0462, P<0.0001; among them P-values are <0.0001, except religion, P = 0.0011 and club, P = 0.0169. Not significant variables are happiness and depression. For live alone or not as the dependent variable significant associations are: bidirectional–all except happiness and likelihood of depression. On the whole, women who do not live alone have more favorable circumstances than those who live alone—happiness, emotional being, satisfaction with life, quality of life and social support, less likelihood of depression, and a higher income.

Summary for the five clusters: each of the 13 variables was analyzed as the dependent (outcome) and as the independent (predictor) variable. Results of Multivariate linear regression show that significant associations, with few exceptions, are bidirectional (Table 3).

CAVEAT: Based on Multivariate linear regression analyses from data of large sample size (n = 93676), interpretations of results may need more attention than from P-values alone. Scrutiny of standard errors, parameter estimates and Partial and Total R-Sqs. may be indicated (Table 3).

## Discussion

Economists, sociologists, psychologists, epidemiologists and others have produced an innumerable number of papers on income and its associations with subjective well-being, health, demographic and psychosocial other factors, and to a lesser extent with religion and social networks such as clubs and lodges. Much of the literature been spawned by early papers [12, 13,14]. Women are rarely included in the extant literature.

This study examined data from relatively healthy women ages 49–79, from a range of race/ethnic groups—Non-white 17% and White 83% of the sample of 93,676 women. It focused on the following variables: income, education, occupation—professional/managerial job—living alone/not alone, general health, likelihood of depression, religion (strength and comfort from), club attendance, and five subjective well-being variables—happiness, emotional well-being, satisfaction with life, quality of life, and social support.

New insights emerged on income, subjective well-being, demographics, behavioral factors, and social networks, here denoted as religion/religiosity and club attendance. Religiosity is measured as ‘strength and comfort from religion’ classified as ‘none’, ‘a little’ and ‘a great deal’. Religion is important in the lives of women irrespective of denomination; 62.98% reported ‘a great deal’; 24.01%; ‘a little’; 12.51%; ‘none’, and 0.5% ‘missing.’ Second is attendance at clubs/lodge/groups, categorized—none, 43.89%; monthly, 40.19%; weekly or more, 13.84%; missing, 1.4%. The two constructs are highly correlated. And, both are associated with subjective well-being.

But, higher income and professional/ managerial job are associated with less strength and comfort from religion, findings are in accord with economist’s studies of income and religion that are primarily based on countrywide data. [4, 13]. However, income and club attendance are not associated, although club attendance and professional/managerial job are.

Both religion and clubs are significantly positively associated with subjective well-being—happiness, emotional well-being, satisfaction with life, quality of life and social support. Poorer health is associated with more from religion, less club attendance; and likelihood of depression (depressed)—less from religion and less club attendance. These findings indicate the importance of religious and club attendance among those who reported ‘better lives’, and their roles in health and well-being. To my knowledge, the effects of club attendance (social networks) on income and subjective well-being have received little in the economic literature.

It may be a cliché to view health is a major concern among virtually of everyone, including relatively healthy women ages 49 to79— 57.92% rated their general health—excellent/ very good, 41.38% good/fair/poor, and 0.70% missing. As noted above, poorer health and more from religion were associated. Poorer health and living alone were also associated.

Emerson wrote: “The first wealth is health”. New observations from this study agree. Quality of life is more highly correlated with general health than any other variable (including other well-being measures). Multivariate linear regression analyses show that foremost predictors are: quality of life of general health, and general health (second to satisfaction with life, a construct similar to quality of life, both of which are based on Cantril’s ladder, and are highly correlated). Income was a minor predictor of general health and of quality of life. Contrary to a common opinion of disparities by socioeconomic status, including depression, in this study likelihood of depression and income were not significantly associated in Multivariate analyses, results in accord with recent studies. [15,16] In conclusion, this study contributes new knowledge relating to income and subjective well-being. Among relatively healthy U.S. women, 17% Non-White and 83% White, ages 49–79, income is not a major factor for emotional well-being happiness, and satisfaction with life; and only marginally, quality of life or social support. Predictors of income in addition to, not surprisingly, education, professional/managerial job, and not living alone are health, religion and quality of life. The finding of the important role that religion and club attendance play in the lives of women suggests the need validation in other populations. The question why is quality of life more highly correlated with general health than other correlations (r = 0.30772), and the primary predictor in multivariate regression analysis. The close relation between satisfaction with life and quality of life (both related to Cantril’s ladder) and the different affects observed here. In this study, definitions and measures used are discussed in detail, in accord with ‘confusion abounds’ in definitions and measurements [2] and ‘Objectivizing the Subjective: Measuring Subjective Wellbeing’ [1]. Hence, it is noteworthy that clarification would contribute to interpretation and implications of research results.

A possible limitation of this study is that the data are from a cross-sectional observational study, which may not be sufficient for analyzing changes over time or causal inference. The strengths of this study are the large sample size and reliability and validity of the questionnaire.

Whether these findings are generalizable globally to diverse populations and a range of demographics–including age, gender, culture, socioeconomics, psychosocial, among others–raise important questions in search of answers. Research by economists, sociologists, psychologists, epidemiologists and others is suggested.

## Supporting Information

S1 Appendix (DOCX)

S1 Table Linear Regression. (DOCX)

S2 Table Manova Results. (DOCX)
