# Supplementary material for: Income and Subjective Well-Being: New Insights from Relatively Healthy American Women, Ages 49-79
Source: PLoS One. 2016 Feb 1;11(2):e0146303. doi: 10.1371/journal.pone.0146303 (PMC4734692; doi:10.1371/journal.pone.0146303)
Supplement: S1 Appendix — (DOCX) [file pone.0146303.s001.docx]

S1 Appendix

In addition to the citations in the text’s Introduction other citations are in this Appendix. Studies are characterized by great differences in methods, measurements, interpretation of empirical results and meta-analyses, as well as views on how and what to study [1-12]**.** Others [13-17] focus primarily on subjective well-being.

On the issue of how best to measure life evaluation varies by author. A terse description is: ‘confusion abounds’ [1]. More research and focus on personal factors and life circumstances are needed [10, 11]. It is concluded that individual well-being is positively linked to absolute income [1].

With respect to income, it was found that individual well-being is positively linked to absolute income 18] and from surveys of subjective well-being a key role for absolute income [19]; the effect of income ‘as comparison income’ was analyzed [20].

Further studies of subjective well-being are: “Subjective well-being is no great issue in sociology”, but, further, job, marital and life satisfaction as topics in sociology [21]. Well-being has eluded definition and review objective measures of subjective well-being [22].

Other included factors are happiness, social networks, and religion. Happiness, a component of subjective well-being, has been the focus in many publications. [8, 9, 16, 17, 22, 23, 24, 26].

Social networks also have with various definitions, but to a lesser degree [27-29]. Explicitly, clubs are mentioned (in relation to teen agers [30] and religion/religiosity [31].

Religion/religiosity and income are currently subjects of interest among economists. The role of culture and religion is justified in studies of growth development and income-- “Culture is usually thought to influence economic outcomes by affecting personal traits such as honesty and work ethic. Religion is one important dimension of culture.” “Empirical research on the determinants of economic growth has typically neglected the influence of religion.” A further conclusion is: “...it would be valuable to extend our analysis of religiosity at a countrywide level to the behavior of individuals.” [32].

From analyses of religion, income, subjective well-being and happiness among individuals in the US and world samples, religiosity is defined as weekly attendance at religious services and reporting that religion is important in one’s daily life [33].

A study of the effect of income and religiousness, concludes that church membership and attendance seem to be similar to membership and participation in social clubs [34]. A bicausal relation between religion and income has been found [35]. Research on religion as a social determinant of health has been called for [36].

References

1. Kahneman D and Deaton A. High Income Improves Evaluation of Life But not Emotional Well-being. Proceedings of the National Academy of Sciences of the United States of America. 2010; 107:16489-6493.
2. Clark E, Kristensen N and Westergård-Nielsen N. Economic Satisfaction and Income Rank in Small Neighbourhoods. Paris School of Economics, Working Paper Num. 2008-44. <halshs-00586254>.
3. Deaton A. Income, Health and Well-being Around the World: Evidence from the Gallup World Poll. Journal of Economic Perspectives*.* April 1 2008; 22(2): 53–72.
4. Diener E. Subjective Well-Being. Psychological Bulletin. 1984: Vol. 95, No. 3, 542-575.
5. Diener E, Biswas-Diener R. Will money increase subjective well-being? Social Indicators Research*.* 2002; 57:119–169.
6. Howell RT and Howell CJ. The relation of economic status to subjective well-being in developing countries: A meta-analysis. Psychological Bulletin*.* 2008; 134:536–560.
7. Ng W, Diener E, Raksha A, Harter J. Affluence, Feelings of Stress, and Well-being. Social Indicators Research*.* 2009: [Volume 94, Number 2](http://www.springerlink.com.ezp-prod1.hul.harvard.edu/content/0303-8300/94/2/), 257-271.
8. Kahneman D, Krueger AB, Schkade D, Schwarz N, Stone AA. Would you be happier if you were richer? A focusing illusion. Science. Jun 30, 2006; 312(5782):1908-10.
9. Wilson W. Correlates of avowed happiness. Psychological Bulletin*.* 1967; 67, 294-306.
10. [Easterlin RA](http://www.ncbi.nlm.nih.gov.ezp-prod1.hul.harvard.edu/pubmed?term=%22Easterlin%20RA%22%5BAuthor%5D). Explaining happiness. Proceedings of the National Academy of Sciences of the United States of America*.* Sep 16, 2003; 100(19):11176-83. Epub 2003 Sep 4.
11. [Easterlin RA](http://www.ncbi.nlm.nih.gov.ezp-prod1.hul.harvard.edu/pubmed?term=%22Easterlin%20RA%22%5BAuthor%5D), [McVey LA](http://www.ncbi.nlm.nih.gov.ezp-prod1.hul.harvard.edu/pubmed?term=%22McVey%20LA%22%5BAuthor%5D), [Switek M](http://www.ncbi.nlm.nih.gov.ezp-prod1.hul.harvard.edu/pubmed?term=%22Switek%20M%22%5BAuthor%5D), [Sawangfa O](http://www.ncbi.nlm.nih.gov.ezp-prod1.hul.harvard.edu/pubmed?term=%22Sawangfa%20O%22%5BAuthor%5D), [Zweig JS](http://www.ncbi.nlm.nih.gov.ezp-prod1.hul.harvard.edu/pubmed?term=%22Zweig%20JS%22%5BAuthor%5D). The happiness–income paradox revisited. Proceedings of the National Academy of Sciences of the United States of America*.* Dec 28, 2010; 107(52):22463-8. Epub 2010 Dec 13.
12. Morrison M, Tay L, Diener E. [Subjective well-being and national satisfaction: findings from a worldwide survey.](http://www.ncbi.nlm.nih.gov.ezp-prod1.hul.harvard.edu/pubmed/21228133) Psychological [Science](http://www.google.com/url?sa=t&rct=j&q=&esrc=s&source=web&cd=1&cad=rja&uact=8&ved=0CCEQFjAA&url=http%3A%2F%2Fpss.sagepub.com%2F&ei=xKgdVdPFCqOwsASC6oCIBA&usg=AFQjCNFrsiDrb3cvtJ2Yr14YOQou5trsLQ&bvm=bv.89744112,d.aWw). Feb 2011; 22(2):166-71. Epub 2011 Jan 12.
13. Clark AE, Frijters P, Shields M. Relative income, happiness and utility: An explanation for the Easterlin paradox and other puzzles. Journal of Economic Literature. 2008; 46:95–144.
14. Headey B, Muffels R, Wooden M. Money does not buy happiness: Or does it? A reassessment based on the combined effects of wealth, income and consumption. Social Indicators Research*.* 2008; 87:65–82.
15. [Quoidbach J](http://www.ncbi.nlm.nih.gov.ezp-prod1.hul.harvard.edu/pubmed?term=%22Quoidbach%20J%22%5BAuthor%5D), [Dunn EW](http://www.ncbi.nlm.nih.gov.ezp-prod1.hul.harvard.edu/pubmed?term=%22Dunn%20EW%22%5BAuthor%5D), [Petrides KV](http://www.ncbi.nlm.nih.gov.ezp-prod1.hul.harvard.edu/pubmed?term=%22Petrides%20KV%22%5BAuthor%5D), [Mikolajczak M](http://www.ncbi.nlm.nih.gov.ezp-prod1.hul.harvard.edu/pubmed?term=%22Mikolajczak%20M%22%5BAuthor%5D). Money giveth, money taketh away: the dual effect of wealth on happiness. Psychological [Science](http://www.google.com/url?sa=t&rct=j&q=&esrc=s&source=web&cd=1&cad=rja&uact=8&ved=0CCEQFjAA&url=http%3A%2F%2Fpss.sagepub.com%2F&ei=xKgdVdPFCqOwsASC6oCIBA&usg=AFQjCNFrsiDrb3cvtJ2Yr14YOQou5trsLQ&bvm=bv.89744112,d.aWw)*.* 2010*;* 21(6):759-63. Epub May 18.
16. Veenhoven R. Hagerty M. Rising happiness in nations, 1946–2.004: A reply to Easterlin. Social Indicators Research*.* 2006; 79:421–436.
17. Veenhoven R. Is happiness Relative? Social Indicators Research*.* 1991; 24:1-34.
18. Frank RH. The Easterlin Paradox Revisited. Emotion. Dec, 2012; 12(6):1188-91. doi: 10.1037/a0029969.Epub 2012 Oct 22.
19. Sacks DW, Stevenson B and Wolfers J. The New Stylized Facts About Income and Subjective Well-Being. Emotion*.* 2012; Vol. 12, No. 6, 1181–1187.
20. Ferrer-i-Carbonell A. Income and well-being: an empirical analysis of the comparison income effect. Journal of Public Economics. Volume 89, Issues 5–6, June, 2005. Pages 997–1019.
21. Veenhoven R. Sociological Theories of Subjective Well-being. (2008). In: Michael Eid & Randy Larsen (Eds). "The Science of Subjective Well-being: A tribute to Ed Diener", Guilford Publications, New York. ISBN 978-1-59385-581-9. pp. 44-61.2008.
22. Wilson WR. An attempt to determine some correlates and dimensions of hedonic tone. (Doctoral dissertation, Northwestern University, 1960)*.* Dissertation Abstracts, 22, 814.
23. Frey BS and Stutzer A. What Can Economists Learn from Happiness Research? Journal of Economic Literature Vol. XL June, 2002. pp. 402–435.
24. Commins RA. Measuring Population Happiness to Inform Public Policy. The 3rd OECD World Forum on “Statistics, Knowledge and Policy”. Busan, Korea - 27-30 October, 2009.
25. Deaton A and Stone AA. Economic Analysis of Subjective Well-being. Two Happiness Puzzles. American Economic Review: Papers & Proceedings. 2013;103(3): 591–597.
26. Frey BS and Stutzer A. What Can Economists Learn from Happiness Research? Journal of Economic Literature Vol. XL June, 2002. pp. 402–435.
27. Commins RA. Measuring Population Happiness to Inform Public Policy. The 3rd OECD World Forum on “Statistics, Knowledge and Policy”. Busan, Korea - 27-30 October, 2009.
28. Deaton A and Stone AA. Economic Analysis of Subjective Well-being. Two Happiness Puzzles. American Economic Review: Papers & Proceedings. 2013;103(3): 591–597.
29. Pinquart M and Sorensen S. Influences of Socioeconomic Status, Social Network, and Competence on Subjective Well-Being in Later Life: A Meta-Analysis. Psychology and Aging. 2009; Vol. 15, No. 2. 187-224.
30. Humpert S. Gender Differences in Life Satisfaction and Social Participation. Leuphana University of Lueneburg (Germany), Institute of [Economics. humpert@leuphana.de](mailto:Economics.%20humpert@leuphana.de). May, 2013.
31. Von Hippel W, Henry JD, Matovic D. Aging and social satisfaction: offsetting positive and negative effects. Psychological Ageing*.* Jun 2008; 23(2):435-9. doi: 10.1037/0882-7974.23.2.435.
32. Barro RJ and McCleary RM. Religion and Economic Growth across Countries. American Sociological Review. 2003; 68, no. 5: 760-781.
33. Diener E, Tay L, Myers DG. The Religion Paradox: if Religion Makes People Happy, Why Are So Many Dropping Out? Journal of Personality and Social Psychology*,* Dec, 2001; 101(6):1278-90. Epub 2011 Aug 1.
34. Buser T. The Effect of Income on Religiousness. CESIFO Working Paper No. 4801CATEGORY 13: BEHAVIOURAL ECONOMICS*.* May 2014.
35. Bettendorf L and Dijkgraaf E. The bicausal relation between religion and income. Applied Economics, Taylor & Francis Journals. 2011; vol. 43(11), pages 1351-1363.
36. Maselko J, Hughes C, Cheney R. Religious social capital: its measurement and utility in the study of the social determinants of health. Social Science and Medicine. Sep 2011; 73(5):759-67. doi: 1016/j.socscimed.2011.06.019. Epub 2011 Jul 12.

|  |  |  |  |  |  |  |
| --- | --- | --- | --- | --- | --- | --- |
