# Supplementary material for: Income and Subjective Well-Being: New Insights from Relatively Healthy American Women, Ages 49-79
Source: PLoS One. 2016 Feb 1;11(2):e0146303. doi: 10.1371/journal.pone.0146303 (PMC4734692; doi:10.1371/journal.pone.0146303)
Supplement: S1 Table — (DOCX) [file pone.0146303.s002.docx]

**S1 Table 1 Linear Regression**

| **Income** |  |  |  |  |  |  | **Education** |  |  |  |  |  |  |
| --- | --- | --- | --- | --- | --- | --- | --- | --- | --- | --- | --- | --- | --- |
|  |  | **Analysis of Variance** | |  |  |  |  |  | **Analysis of Variance** | |  |  |  |
| **Source** | **DF** | **Sum of** | **Mean** | **F Value** | **Pr > F** |  | **Source** | **DF** | **Sum of** | **Mean** | **F Value** | **Pr > F** |  |
|  |  | **Squares** | **Square** |  |  |  |  |  | **Squares** | **Square** |  |  |  |
| **Model** | **Model** | 12 | 36362 | 3030.134 | 1215.51 |  | **Model** | 12 | 12306 | 1025.50252 | 2762.27 | <.0001 |  |
| **Error** | **Error** | 86779 | 216331 | 2.49289 |  |  | **Error** | 86779 | 32217 | 0.37125 |  |  |  |
| **Corrected Total** | **Corrected Total** | 86791 | 252692 |  |  |  | **Corrected Total** | 86791 | 44523 |  |  |  |  |
| **Root MSE** | 1.57888 | **R-Square** | 0.1439 |  |  |  | **Root MSE** | 0.60931 | **R-Square** | 0.2764 |  |  |  |
| **Dependent Mean** | 4.15368 | **Adj R-Sq** | 0.1438 |  |  |  | **Dependent Mean** | 2.09388 | **Adj R-Sq** | 0.2763 |  |  |  |
| **Coeff Var** | 38.0117 |  |  |  |  |  | **Coeff Var** | 29.09938 |  |  |  |  |  |
|  |  |  |  |  |  |  |  |  |  |  |  |  |  |
| **Parameter Estimates** | |  |  |  |  |  | **Parameter Estimates** | |  |  |  |  |  |
| **Variable** | **DF** | **Parameter** | **Standard** | **t Value** | **Pr > \|t\|** |  | **Variable** | **DF** | **Parameter** | **Standard** | **t Value** | **Pr > \|t\|** |  |
|  |  | **Estimate** | **Error** |  |  |  |  |  | **Estimate** | **Error** |  |  |  |
| **Intercept** | 1 | 2.2508 | 0.0423 | 53.2400 | <.0001 |  | **Intercept** | **1** | 0.9539 | 0.0163 | 58.67 | <.0001 |  |
| **Education** | 1 | 0.6816 | 0.0085 | 80.3200 | <.0001 |  | **Income** | **1** | 0.1015 | 0.0013 | 80.32 | <.0001 |  |
| **Clubs** | 1 | 0.0085 | 0.0075 | 1.1300 | 0.2604 |  | **Clubs** | **1** | 0.0019 | 0.0029 | 0.65 | 0.5177 |  |
| **Religion** | 1 | -0.0385 | 0.0075 | -5.1400 | <.0001 |  | **Religion** | **1** | -0.0048 | 0.0029 | -1.66 | 0.0964 |  |
| **Health** | 1 | 0.0509 | 0.0114 | 4.4500 | <.0001 |  | **Health** | **1** | -0.0027 | 0.0044 | -0.62 | 0.5359 |  |
| **Depression** | 1 | 0.00000138 | 0.0110 | 0.0000 | 0.9999 |  | **Depression** | **1** | -0.0024 | 0.0043 | -0.57 | 0.5675 |  |
| **Live Alone** | 1 | -0.1515 | 0.0121 | -12.5300 | <.0001 |  | **Live Alone** | **1** | 0.0194 | 0.0047 | 4.14 | <.0001 |  |
| **Job** | 1 | 0.4290 | 0.0107 | 40.0700 | <.0001 |  | **Job** | **1** | 0.5098 | 0.0038 | 134.4 | <.0001 |  |
| **Happy** | 1 | -0.0013 | 0.0067 | -0.1900 | 0.8462 |  | **Happy** | **1** | 0.0001 | 0.0026 | 0.03 | 0.9781 |  |
| **Well-being** | 1 | -0.0096 | 0.0087 | -1.1000 | 0.2713 |  | **Well-being** | **1** | 0.0050 | 0.0034 | 1.49 | 0.1362 |  |
| **Satisfied w/Life** | 1 | -0.0086 | 0.0168 | -0.5100 | 0.6074 |  | **Satisfied w/Life** | **1** | -0.0046 | 0.0065 | -0.7 | 0.4823 |  |
| **Quality of Life** | 1 | 0.0606 | 0.0167 | 3.6400 | 0.0003 |  | **Quality of Life** | **1** | 0.0027 | 0.0064 | 0.42 | 0.6757 |  |
| **Social Support** | 1 | 0.0199 | 0.0108 | 1.8500 | 0.0647 |  | **Social Support** | **1** | 0.0005 | 0.0042 | 0.13 | 0.8900 |  |
|  |  |  |  |  |  |  |  |  |  |  |  |  |  |
| **Religion** |  |  |  |  |  |  | **Clubs** |  |  |  |  |  |  |
| **Analysis of Variance** | | | | | |  | **Analysis of Variance** | | | | | |  |
| **Source** | **DF** | **Sum of** | **Mean** | **F Value** | **Pr > F** |  | **Source** | **DF** | **Sum of** | **Mean** | **F Value** | **Pr > F** |  |
|  |  | **Squares** | **Square** |  |  |  |  |  | **Squares** | **Square** |  |  |  |
| **Model** | 12 | 1691.2516 | 140.93763 | 275.25 | <.0001 |  | **Model** | 12 | 1316.75774 | 109.72981 | 215.49 | <.0001 |  |
| **Error** | 86779 | 44433 | 0.51203 |  |  |  | **Error** | 86779 | 44188 | 0.50921 |  |  |  |
| **Corrected Total** | 86791 | 46124 |  |  |  |  | **Corrected Total** | 86791 | 45505 |  |  |  |  |
| **Root MSE** | 0.71556 | **R-Square** | 0.0367 |  |  |  | **Root MSE** | 0.71359 | **R-Square** | 0.0289 |  |  |  |
| **Dependent Mean** | 2.49352 | **Adj R-Sq** | 0.0365 |  |  |  | **Dependent Mean** | 1.67184 | **Adj R-Sq** | 0.0288 |  |  |  |
| **Coeff Var** | 28.69673 |  |  |  |  |  | **Coeff Var** | 42.68286 |  |  |  |  |  |
|  |  |  |  |  |  |  |  |  |  |  |  |  |  |
| **Parameter Estimates** | | | | | |  | **Parameter Estimates** | | | | | |  |
| **Variable** | **DF** | **Parameter** | **Standard** | **t Value** | **Pr > \|t\|** |  | **Variable** | **DF** | **Parameter** | **Standard** | **t Value** | **Pr > \|t\|** |  |
|  |  | **Estimate** | **Error** |  |  |  |  |  | **Estimate** | **Error** |  |  |  |
| **Intercept** | **1** | 2.1717 | 0.0180 | 120.52 | <.0001 |  | **Intercept** | **1** | 0.9303 | 0.0192 | 48.56 | <.0001 |  |
| **Income** | **1** | -0.0079 | 0.0015 | -5.14 | <.0001 |  | **Income** | **1** | 0.0017 | 0.0015 | 1.13 | 0.2604 |  |
| **Education** | **1** | -0.0066 | 0.0040 | -1.66 | 0.0964 |  | **Education** | **1** | 0.0026 | 0.0040 | 0.65 | 0.5177 |  |
| **Clubs** | **1** | 0.1060 | 0.0034 | 31.32 | <.0001 |  | **Religion** | **1** | 0.1054 | 0.0034 | 31.32 | <.0001 |  |
| **Health** | **1** | -0.1483 | 0.0052 | -28.76 | <.0001 |  | **Health** | **1** | 0.0380 | 0.0052 | 7.36 | <.0001 |  |
| **Depression** | **1** | -0.0113 | 0.0050 | -2.27 | 0.0232 |  | **Depression** | **1** | 0.0398 | 0.0050 | 8.01 | <.0001 |  |
| **Live Alone** | **1** | 0.0190 | 0.0055 | 3.47 | 0.0005 |  | **Live Alone** | **1** | 0.1048 | 0.0055 | 19.2 | <.0001 |  |
| **Job** | **1** | -0.0111 | 0.0049 | -2.26 | 0.0239 |  | **Job** | **1** | 0.0124 | 0.0049 | 2.55 | 0.0109 |  |
| **Happy** | **1** | 0.0105 | 0.0030 | 3.48 | 0.0005 |  | **Happy** | **1** | -0.0047 | 0.0030 | -1.57 | 0.1158 |  |
| **Well-being** | **1** | 0.0212 | 0.0039 | 5.39 | <.0001 |  | **Well-being** | **1** | 0.0398 | 0.0039 | 10.13 | <.0001 |  |
| **Satisfied w/Life** | **1** | 0.1012 | 0.0076 | 13.29 | <.0001 |  | **Satisfied w/Life** | **1** | 0.0320 | 0.0076 | 4.22 | <.0001 |  |
| **Quality of Life** | **1** | 0.0663 | 0.0076 | 8.78 | <.0001 |  | **Quality of Life** | **1** | 0.0385 | 0.0075 | 5.11 | <.0001 |  |
| **Social Support** | **1** | 0.0618 | 0.0049 | 12.66 | <.0001 |  | **Social Support** | **1** | 0.0178 | 0.0049 | 3.64 | 0.0003 |  |
|  |  |  |  |  |  |  |  |  |  |  |  |  |  |
| **Job** |  |  |  |  |  |  | **Live Alone** |  |  |  |  |  |  |
| **Analysis of Variance** | | | | | |  | **Analysis of Variance** | | | | | |  |
| **Source** | **DF** | **Sum of** | **Mean** | **F Value** | **Pr > F** |  | **Source** | **DF** | **Sum of** | **Mean** | **F Value** | **Pr > F** |  |
|  |  | **Squares** | **Square** |  |  |  |  |  | **Squares** | **Square** |  |  |  |
| **Model** | 12 | 6627.58422 | 552.29869 | 2243.82 | <.0001 |  | **Model** | 12 | 892.42424 | 74.36869 | 379.36 | <.0001 |  |
| **Error** | 86779 | 21360 | 0.24614 |  |  |  | **Error** | 86779 | 17012 | 0.19604 |  |  |  |
| **Corrected Total** | 86791 | 27988 |  |  |  |  | **Corrected Total** | 86791 | 17905 |  |  |  |  |
| **Root MSE** | 0.49613 | **R-Square** | 0.2368 |  |  |  | **Root MSE** | 0.44276 | **R-Square** | 0.0498 |  |  |  |
| **Dependent Mean** | 1.376 | **Adj R-Sq** | 0.2367 |  |  |  | **Dependent Mean** | 1.25533 | **Adj R-Sq** | 0.0497 |  |  |  |
| **Coeff Var** | 36.05568 |  |  |  |  |  | **Coeff Var** | 35.27055 |  |  |  |  |  |
|  |  |  |  |  |  |  |  |  |  |  |  |  |  |
| **Parameter Estimates** | | | | | |  | **Parameter Estimates** | | | | | |  |
| **Variable** | **DF** | **Parameter** | **Standard** | **t Value** | **Pr > \|t\|** |  | **Variable** | **DF** | **Parameter** | **Standard** | **t Value** | **Pr > \|t\|** |  |
|  |  | **Estimate** | **Error** |  |  |  |  |  | **Estimate** | **Error** |  |  |  |
| **Intercept** | **1** | 0.4737 | 0.0134 | 35.34 | <.0001 |  | **Intercept** | **1** | 1.4837 | 0.0109 | 135.58 | <.0001 |  |
| **Income** | **1** | 0.0424 | 0.0011 | 40.07 | <.0001 |  | **Income** | **1** | -0.0119 | 0.0010 | -12.53 | <.0001 |  |
| **Education** | **1** | 0.3380 | 0.0025 | 134.4 | <.0001 |  | **Education** | **1** | 0.0102 | 0.0025 | 4.14 | <.0001 |  |
| **Clubs** | **1** | 0.0060 | 0.0024 | 2.55 | 0.0109 |  | **Clubs** | **1** | 0.0404 | 0.0021 | 19.2 | <.0001 |  |
| **Religion** | **1** | -0.0053 | 0.0024 | -2.26 | 0.0239 |  | **Religion** | **1** | 0.0073 | 0.0021 | 3.47 | 0.0005 |  |
| **Health** | **1** | 0.0072 | 0.0036 | 1.99 | 0.0461 |  | **Health** | **1** | 0.0241 | 0.0032 | 7.52 | <.0001 |  |
| **Depression** | **1** | -0.0019 | 0.0035 | -0.54 | 0.5917 |  | **Depression** | **1** | -0.0027 | 0.0031 | -0.86 | 0.3879 |  |
| **Live Alone** | **1** | 0.0091 | 0.0038 | 2.4 | 0.0165 |  | **Job** | **1** | 0.0073 | 0.0030 | 2.4 | 0.0165 |  |
| **Happy** | **1** | 0.0005 | 0.0021 | 0.25 | 0.8064 |  | **Happy** | **1** | -0.0338 | 0.0019 | -18.17 | <.0001 |  |
| **Well-being** | **1** | 0.0023 | 0.0027 | 0.85 | 0.3938 |  | **Well-being** | **1** | 0.0521 | 0.0024 | 21.45 | <.0001 |  |
| **Satisfied w/Life** | **1** | -0.0033 | 0.0053 | -0.63 | 0.5298 |  | **Satisfied w/Life** | **1** | -0.0127 | 0.0047 | -2.68 | 0.0073 |  |
| **Quality of Life** | **1** | -0.0029 | 0.0052 | -0.55 | 0.5848 |  | **Quality of Life** | **1** | -0.0390 | 0.0047 | -8.35 | <.0001 |  |
| **Social Support** | **1** | 0.0025 | 0.0034 | 0.75 | 0.4544 |  | **Social Support** | **1** | -0.1498 | 0.0030 | -50.29 | <.0001 |  |
|  |  |  |  |  |  |  |  |  |  |  |  |  |  |
| **Happy** |  |  |  |  |  |  | **WellBeing** |  |  |  |  |  |  |
| **Analysis of Variance** | | | | | |  | **Analysis of Variance** | | | | | |  |
| **Source** | **DF** | **Sum of** | **Mean** | **F Value** | **Pr > F** |  | **Source** | **DF** | **Sum of** | **Mean** | **F Value** | **Pr > F** |  |
|  |  | **Squares** | **Square** |  |  |  |  |  | **Squares** | **Square** |  |  |  |
| **Model** | 12 | 37982 | 3165.13618 | 4877.56 | <.0001 |  | **Model** | 12 | 30757 | 2563.103 | 6735.39 | <.0001 |  |
| **Error** | 86779 | 56312 | 0.64892 |  |  |  | **Error** | 86779 | 33023 | 0.38054 |  |  |  |
| **Corrected Total** | 86791 | 94294 |  |  |  |  | **Corrected Total** | 86791 | 63780 |  |  |  |  |
|  |  |  |  |  |  |  |  |  |  |  |  |  |  |
| **Root MSE** | 0.80555 | **R-Square** | 0.4028 |  |  |  | **Root MSE** | 0.61688 | **R-Square** | 0.4822 |  |  |  |
| **Dependent Mean** | 4.5624 | **Adj R-Sq** | 0.4027 |  |  |  | **Dependent Mean** | 2.2403 | **Adj R-Sq** | 0.4822 |  |  |  |
| **Coeff Var** | 17.65636 |  |  |  |  |  | **Coeff Var** | 27.53567 |  |  |  |  |  |
| **Parameter Estimates** | | | | | |  | **Parameter Estimates** | | | | | |  |
| **Variable** | **DF** | **Parameter** | **Standard** | **t Value** | **Pr > \|t\|** |  | **Variable** | **DF** | **Parameter** | **Standard** | **t Value** | **Pr > \|t\|** |  |
|  |  | **Estimate** | **Error** |  |  |  |  |  | **Estimate** | **Error** |  |  |  |
| **Intercept** | **1** | 2.554 | 0.020 | 126.89 | <.0001 |  | **Intercept** | **1** | -0.9518 | 0.0165 | -57.79 | <.0001 |  |
| **Income** | **1** | 0.000 | 0.002 | -0.19 | 0.8462 |  | **Income** | **1** | -0.0015 | 0.0013 | -1.1 | 0.2713 |  |
| **Education** | **1** | 0.000 | 0.004 | 0.03 | 0.9781 |  | **Education** | **1** | 0.0051 | 0.0034 | 1.49 | 0.1362 |  |
| **Clubs** | **1** | -0.006 | 0.004 | -1.57 | 0.1158 |  | **Clubs** | **1** | 0.0297 | 0.0029 | 10.13 | <.0001 |  |
| **Religion** | **1** | 0.013 | 0.004 | 3.48 | 0.0005 |  | **Religion** | **1** | 0.0158 | 0.0029 | 5.39 | <.0001 |  |
| **Health** | **1** | 0.017 | 0.006 | 2.96 | 0.0031 |  | **Health** | **1** | 0.1727 | 0.0044 | 39.02 | <.0001 |  |
| **Depression** | **1** | 0.065 | 0.006 | 11.6 | <.0001 |  | **Depression** | **1** | 0.3689 | 0.0041 | 89.61 | <.0001 |  |
| **Live Alone** | **1** | 0.001 | 0.006 | 0.25 | 0.8064 |  | **Live Alone** | **1** | 0.0036 | 0.0042 | 0.85 | 0.3938 |  |
| **Job** | **1** | -0.112 | 0.006 | -18.17 | <.0001 |  | **Job** | **1** | 0.1012 | 0.0047 | 21.45 | <.0001 |  |
| **Well-being** | **1** | 0.633 | 0.004 | 163.11 | <.0001 |  | **Happy** | **1** | 0.3709 | 0.0023 | 163.11 | <.0001 |  |
| **Satisfied w/Life** | **1** | 0.195 | 0.009 | 22.82 | <.0001 |  | **Satisfied w/Life** | **1** | 0.1408 | 0.0066 | 21.49 | <.0001 |  |
| **Quality of Life** | **1** | 0.101 | 0.008 | 11.92 | <.0001 |  | **Quality of Life** | **1** | 0.0692 | 0.0065 | 10.65 | <.0001 |  |
| **Social Support** | **1** | 0.099 | 0.005 | 17.96 | <.0001 |  | **Social Support** | **1** | 0.0923 | 0.0042 | 21.97 | <.0001 |  |
|  |  |  |  |  |  |  |  |  |  |  |  |  |  |
| **Satisfied w/Life** |  |  |  |  |  |  | **Quality of Life** |  |  |  |  |  |  |
| **Analysis of Variance** | | | | | |  | **Analysis of Variance** | | | | | |  |
| **Source** | **DF** | **Sum of** | **Mean** | **F Value** | **Pr > F** |  | **Source** | **DF** | **Sum of** | **Mean** | **F Value** | **Pr > F** |  |
|  |  | **Squares** | **Square** |  |  |  |  |  | **Squares** | **Square** |  |  |  |
| **Model** | 12 | 14181 | 1181.77058 | 11625.2 | <.0001 |  | **Model** | 12 | 13961 | 1163.39475 | 11235.7 | <.0001 |  |
| **Error** | 86779 | 8821.61357 | 0.10166 |  |  |  | **Error** | 86779 | 8985.50702 | 0.10354 |  |  |  |
| **Corrected Total** | 86791 | 23003 |  |  |  |  | **Corrected Total** | 86791 | 22946 |  |  |  |  |
| **Root MSE** | 0.31884 | **R-Square** | 0.6165 |  |  |  | **Root MSE** | 0.32178 | **R-Square** | 0.6084 |  |  |  |
| **Dependent Mean** | 1.49399 | **Adj R-Sq** | 0.6164 |  |  |  | **Dependent Mean** | 1.46749 | **Adj R-Sq** | 0.6084 |  |  |  |
| **Coeff Var** | 21.34127 |  |  |  |  |  | **Coeff Var** | 21.92755 |  |  |  |  |  |
|  |  |  |  |  |  |  |  |  |  |  |  |  |  |
| **Parameter Estimates** | | | | | |  | **Parameter Estimates** | | | | | |  |
| **Variable** | **DF** | **Parameter** | **Standard** | **t Value** | **Pr > \|t\|** |  | **Variable** | **DF** | **Parameter** | **Standard** | **t Value** | **Pr > \|t\|** |  |
|  |  | **Estimate** | **Error** |  |  |  |  |  | **Estimate** | **Error** |  |  |  |
| **Intercept** | **1** | 0.0403 | 0.0087 | 4.65 | <.0001 |  | **Intercept** | **1** | 0.0703 | 0.0088 | 8.03 | <.0001 |  |
| **Income** | **1** | -0.0004 | 0.0007 | -0.51 | 0.6074 |  | **Income** | **1** | 0.0025 | 0.0007 | 3.64 | 0.0003 |  |
| **Education** | **1** | -0.0013 | 0.0018 | -0.7 | 0.4823 |  | **Education** | **1** | 0.00075007 | 0.0018 | 0.42 | 0.6757 |  |
| **Clubs** | **1** | 0.0064 | 0.0015 | 4.22 | <.0001 |  | **Clubs** | **1** | 0.0078 | 0.0015 | 5.11 | <.0001 |  |
| **Religion** | **1** | 0.0201 | 0.0015 | 13.29 | <.0001 |  | **Religion** | **1** | 0.0134 | 0.0015 | 8.78 | <.0001 |  |
| **Health** | **1** | 0.0341 | 0.0023 | 14.81 | <.0001 |  | **Health** | **1** | 0.0761 | 0.0023 | 32.86 | <.0001 |  |
| **Depression** | **1** | 0.0311 | 0.0022 | 14 | <.0001 |  | **Depression** | **1** | 0.0085 | 0.0022 | 3.77 | 0.0002 |  |
| **Live Alone** | **1** | -0.0014 | 0.0022 | -0.63 | 0.5298 |  | **Live Alone** | **1** | -0.0012 | 0.0022 | -0.55 | 0.5848 |  |
| **Job** | **1** | -0.0066 | 0.0024 | -2.68 | 0.0073 |  | **Job** | **1** | -0.0206 | 0.0025 | -8.35 | <.0001 |  |
| **Happy** | **1** | 0.0306 | 0.0013 | 22.82 | <.0001 |  | **Happy** | **1** | 0.0162 | 0.0014 | 11.92 | <.0001 |  |
| **Well-being** | **1** | 0.0376 | 0.0018 | 21.49 | <.0001 |  | **Well-being** | **1** | 0.0188 | 0.0018 | 10.65 | <.0001 |  |
| **Quality of Life** | **1** | 0.6813 | 0.0024 | 278.94 | <.0001 |  | **Satisfied w/Life** | **1** | 0.6939 | 0.0025 | 278.94 | <.0001 |  |
| **Social Support** | **1** | 0.0576 | 0.0022 | 26.56 | <.0001 |  | **Social Support** | **1** | 0.0564 | 0.0022 | 25.75 | <.0001 |  |
|  |  |  |  |  |  |  |  |  |  |  |  |  |  |
| **Social Support** |  |  |  |  |  |  |  |  |  |  |  |  |  |
| **Analysis of Variance** | | | | | |  |  |  |  |  |  |  |  |
| **Source** | **DF** | **Sum of** | **Mean** | **F Value** | **Pr > F** |  |  |  |  |  |  |  |  |
|  |  | **Squares** | **Square** |  |  |  |  |  |  |  |  |  |  |
| **Model** | 12 | 4772.07932 | 397.67328 | 1608.38 | <.0001 |  |  |  |  |  |  |  |  |
| **Error** | 86779 | 21456 | 0.24725 |  |  |  |  |  |  |  |  |  |  |
| **Corrected Total** | 86791 | 26228 |  |  |  |  |  |  |  |  |  |  |  |
|  |  |  |  |  |  |  |  |  |  |  |  |  |  |
| **Root MSE** | 0.49724 | **R-Square** | 0.1819 |  |  |  |  |  |  |  |  |  |  |
| **Dependent Mean** | 1.44183 | **Adj R-Sq** | 0.1818 |  |  |  |  |  |  |  |  |  |  |
| **Coeff Var** | 34.48706 |  |  |  |  |  |  |  |  |  |  |  |  |
|  |  |  |  |  |  |  |  |  |  |  |  |  |  |
| **Parameter Estimates** | | | | | |  |  |  |  |  |  |  |  |
| **Variable** | **DF** | **Parameter** | **Standard** | **t Value** | **Pr > \|t\|** |  |  |  |  |  |  |  |  |
|  |  | **Estimate** | **Error** |  |  |  |  |  |  |  |  |  |  |
| **Intercept** | **1** | 0.7167 | 0.0133 | 53.85 | <.0001 |  |  |  |  |  |  |  |  |
| **Income** | **1** | 0.0020 | 0.0011 | 1.85 | 0.0647 |  |  |  |  |  |  |  |  |
| **Education** | **1** | 0.0004 | 0.0028 | 0.13 | 0.899 |  |  |  |  |  |  |  |  |
| **Clubs** | **1** | 0.0086 | 0.0024 | 3.64 | 0.0003 |  |  |  |  |  |  |  |  |
| **Religion** | **1** | 0.0298 | 0.0024 | 12.66 | <.0001 |  |  |  |  |  |  |  |  |
| **Health** | **1** | 0.0422 | 0.0036 | 11.74 | <.0001 |  |  |  |  |  |  |  |  |
| **Depression** | **1** | 0.0545 | 0.0035 | 15.74 | <.0001 |  |  |  |  |  |  |  |  |
| **Live Alone** | **1** | 0.0026 | 0.0034 | 0.75 | 0.4544 |  |  |  |  |  |  |  |  |
| **Job** | **1** | -0.1890 | 0.0038 | -50.29 | <.0001 |  |  |  |  |  |  |  |  |
| **Happy** | **1** | 0.0376 | 0.0021 | 17.96 | <.0001 |  |  |  |  |  |  |  |  |
| **Well-being** | **1** | 0.0600 | 0.0027 | 21.97 | <.0001 |  |  |  |  |  |  |  |  |
| **Satisfied w/Life** | **1** | 0.1400 | 0.0053 | 26.56 | <.0001 |  |  |  |  |  |  |  |  |
| **Quality of Life** | **1** | 0.1346 | 0.0052 | 25.75 | <.0001 |  |  |  |  |  |  |  |  |
|  |  |  |  |  |  |  |  |  |  |  |  |  |  |
| Note: |  |  |  |  |  |  |  |  |  |  |  |  |  |
| **F= Mean Square of the Independent Variable/Mean Square Error of the Dependent Variable.** | | | | | | | |  |  |  |  |  |  |
| **Pr>F in effect P value** | |  |  |  |  |  |  |  |  |  |  |  |  |
| **t value--Parameter Estimate/Standard Error** | | |  |  |  |  |  |  |  |  |  |  |  |
