# Supplementary material for: Income and Subjective Well-Being: New Insights from Relatively Healthy American Women, Ages 49-79
Source: PLoS One. 2016 Feb 1;11(2):e0146303. doi: 10.1371/journal.pone.0146303 (PMC4734692; doi:10.1371/journal.pone.0146303)
Supplement: S2 Table — (DOCX) [file pone.0146303.s003.docx]

**S2 Table 1 Manova Results**

| **Income** |  |  |  |  |  |  | **Education** |  |  |  |  |  |  |
| --- | --- | --- | --- | --- | --- | --- | --- | --- | --- | --- | --- | --- | --- |
| **Source** | **DF** | **Sum of Sqs** | **Mean Square** | **F Value*** | **Pr > F** |  | **Source** | **DF** | **Sum of Sqs** | **Mean Square** | **F Value*** | **Pr > F** |  |
| **Model** | 31 | 36722.55 | 1184.60 | 475.88 | <.0001 |  | **Model** | 36 | 14072.06 | 390.89 | 1113.65 | <.0001 |  |
| **Error** | 86760 | 215969.70 | 2.49 |  |  |  | **Error** | 86755 | 30451.01 | 0.351 |  |  |  |
| **Corrected Total** | 86791 | 252692.25 |  |  |  |  | **Corrected Total** | 86791 | 44523.07 |  |  |  |  |
| **R-Square** | **Coeff Var** | **Root MSE** | **Mean** |  |  |  | **R-Square** | **Coeff Var** | **Root MSE** | **Mean** |  |  |  |
| 0.1453 | 37.98 | 1.58 | 4.15 |  |  |  | 0.3161 | 28.29 | 0.59 | 2.09 |  |  |  |
| **Source** | **DF** | **Type III SS** | **Mean Square** | **F Value**** | **Pr > F** |  | **Source** | **DF** | **Type III SS** | **Mean Square** | **F Value**** | **Pr > F** |  |
| **Education** | 2 | 14577.53 | 7288.76 | 2928.06 | <.0001 |  | **Income** | 7 | 2108.76 | 301.25 | 858.27 | <.0001 |  |
| **Clubs** | 3 | 6.38 | 2.13 | .85 | .4643 |  | **Clubs** | 3 | 2.61 | .87 | 2.48 | .0593 |  |
| **Religion** | 3 | 79.40 | 26.47 | 10.63 | <.0001 |  | **Religion** | 3 | 2.81 | .94 | 2.67 | .0459 |  |
| **Health** | 2 | 51.93 | 25.97 | 10.43 | <.0001 |  | **Health** | 2 | .02 | .01 | .03 | .9688 |  |
| **Depressed** | 2 | .94 | .47 | .19 | .8280 |  | **Depressed** | 2 | 1.26 | .63 | 1.80 | .1659 |  |
| **Live Alone** | 2 | 408.31 | 204.16 | 82.01 | <.0001 |  | **Live Alone** | 2 | 4.02 | 2.01 | 5.72 | .0033 |  |
| **Job** | 2 | 4274.72 | 2137.36 | 858.63 | <.0001 |  | **Job** | 2 | 8208.71 | 4104.35 | 11693.30 | <.0001 |  |
| **Happy** | 6 | 15.07 | 2.51 | 1.01 | .4170 |  | **Happy** | 6 | .57 | .10 | .27 | .9503 |  |
| **Well-being** | 3 | 6.24 | 2.08 | .84 | .4741 |  | **Well-being** | 3 | 1.69 | .56 | 1.61 | .1854 |  |
| **Satisfied w/life** | 2 | .93 | .46 | .19 | .8302 |  | **Satisfied w/life** | 2 | .20 | .10 | .28 | .7542 |  |
| **Quality of Life** | 2 | 35.38 | 17.69 | 7.11 | .0008 |  | **Quality of Life** | 2 | .39 | .19 | .56 | .5738 |  |
| **Social Support** | 2 | 4.80 | 2.40 | .96 | .3814 |  | **Social Support** | 2 | 2.11 | 1.05 | 3.00 | .0497 |  |
|  |  |  |  |  |  |  |  |  |  |  |  |  |  |
| **Religion Gives Strength and Comfort** | | |  |  |  |  | **Attend clubs/lodges/groups** | |  |  |  |  |  |
| **Source** | **DF** | **Sum of Sqs** | **Mean Square** | **F Value*** | **Pr > F** |  | **Source** | **DF** | **Sum of Sqs** | **Mean Square** | **F Value*** | **Pr > F** |  |
| **Model** | 35 | 2522.19 | 72.063 | 143.38 | <.0001 |  | **Model** | 35 | 1636.451 | 46.75575 | 92.47 | <.0001 |  |
| **Error** | 86756 | 43602.17 | 0.503 |  |  |  | **Error** | 86756 | 43868.785 | 0.50566 |  |  |  |
| **Corrected Total** | 86791 | 46124.36 |  |  |  |  | **Corrected Total** | 86791 | 45505.236 |  |  |  |  |
| **R-Square** | **Coeff Var** | **Root MSE** | **Mean** |  |  |  | **R-Square** | **Coeff Var** | **Root MSE** | **Mean** |  |  |  |
| 0.0547 | 28.4309 | 0.7089 | 2.4935 |  |  |  | 0.0360 | 42.534 | 0.711 | 1.672 |  |  |  |
| **Source** | **DF** | **Type III SS** | **Mean Square** | **F Value**** | **Pr > F** |  | **Source** | **DF** | **Type III SS** | **Mean Square** | **F Value**** | **Pr > F** |  |
| **Income** | 7 | 15.46 | 2.21 | 4.40 | <.0001 |  | **Income** | 7 | 1.74 | 0.25 | 0.49 | 0.8412 |  |
| **Education** | 2 | 0.75 | 0.37 | 0.74 | 0.4764 |  | **Education** | 2 | 0.49 | 0.25 | 0.49 | 0.6145 |  |
| **Clubs** | 3 | 427.76 | 142.59 | 283.70 | <.0001 |  | **Religion** | 3 | 436.20 | 145.40 | **287.54** | <.0001 |  |
| **Health** | 2 | 596.46 | 298.23 | 593.39 | <.0001 |  | **Health** | 2 | 8.34 | 4.17 | 8.24 | 0.0003 |  |
| **Depressed** | 2 | 15.29 | 7.64 | 15.21 | <.0001 |  | **Depressed** | 2 | 43.20 | 21.60 | 42.72 | <.0001 |  |
| **Live alone** | 2 | 172.99 | 86.49 | 172.10 | <.0001 |  | **Live alone** | 2 | 139.15 | 69.57 | **137.59** | <.0001 |  |
| **Job** | 2 | 3.98 | 1.99 | 3.96 | 0.0191 |  | **Job** | 2 | 3.63 | 1.82 | 3.59 | 0.0275 |  |
| **Happy** | 6 | 216.73 | 36.12 | 71.87 | <.0001 |  | **Happy** | 6 | 56.74 | 9.46 | 18.70 | <.0001 |  |
| **Well-Being** | 3 | 12.75 | 4.25 | 8.46 | <.0001 |  | **Well-Being** | 3 | 23.10 | 7.70 | 15.23 | <.0001 |  |
| **Satisfied w/life** | 2 | 51.75 | 25.88 | 51.49 | <.0001 |  | **Satisfied w/life** | 2 | 7.48 | 3.74 | 7.40 | 0.0006 |  |
| **Quality of Life** | 2 | 22.31 | 11.15 | 22.19 | <.0001 |  | **Quality of Life** | 2 | 10.02 | 5.01 | 9.90 | <.0001 |  |
| **Social Support** | 2 | 28.05 | 14.03 | 27.91 | <.0001 |  | **Social Support** | 2 | 1.10 | 0.55 | 1.09 | **0.3371** |  |
|  |  |  |  |  |  |  |  |  |  |  |  |  |  |
| **Managerial/Professional Job** | |  |  |  |  |  | **Live Alone** |  |  |  |  |  |  |
| **Source** | **DF** | **Sum of Sqs** | **Mean Square** | **F Value*** | **Pr > F** |  | **Source** | **DF** | **Sum of Sqs** | **Mean Square** | **F Value*** | **Pr > F** |  |
| **Model** | 36 | 6979.47 | 193.87 | 800.62 | <.0001 |  | **Model** | 36 | 1383.69 | 38.44 | 201.84 | <.0001 |  |
| **Error** | 86755 | 21008.07 | 0.24215 |  |  |  | **Error** | 86775 | 16520.84 | 0.19043 |  |  |  |
| **Corrected Total** | 86791 | 27987.54 |  |  |  |  | **Corrected Total** | 86791 | 17904.53 |  |  |  |  |
| **R-Square** | **Coeff Var** | **Root MSE** | **Mean** |  |  |  | **R-Square** | **Coeff Var** | **Root MSE** | **Mean** |  |  |  |
| .2494 | 35.76 | .49 | 1.38 |  |  |  | .0773 | 34.76 | .44 | 1.26 |  |  |  |
| **Source** | **DF** | **Type III SS** | **Mean Square** | **F Value**** | **Pr > F** |  | **Source** | **DF** | **Type III SS** | **Mean Square** | **F Value**** | **Pr > F** |  |
| **Income** | 7 | 488.10 | 69.73 | 287.95 | <.0001 |  | **Income** | **7** | 31.72 | 4.53 | 23.80 | <.0001 |  |
| **Education** | 2 | 4591.47 | 2295.74 | 9480.48 | <.0001 |  | **Education** | **2** | 2.47 | 1.24 | 6.50 | .0015 |  |
| **Clubs** | 3 | 9.66 | 3.22 | 13.30 | <.0001 |  | **Clubs** | **3** | 55.12 | 18.37 | 96.49 | <.0001 |  |
| **Religion** | 3 | 2.07 | .69 | 2.85 | .0358 |  | **Religion** | **3** | 96.30 | 32.10 | 168.56 | <.0001 |  |
| **Health** | 2 | .64 | .32 | 1.31 | .2686 |  | **Health** | **2** | 2.69 | 1.35 | 7.07 | .0009 |  |
| **Depressed** | 2 | .12 | .06 | .26 | .7738 |  | **Depressed** | **2** | 6.32 | 3.16 | 16.60 | <.0001 |  |
| **Live Alone** | 2 | 1.77 | .88 | 3.65 | .0260 |  | **Job** | **2** | 2.99 | 1.50 | 7.86 | .0004 |  |
| **Happy** | 6 | 2.17 | .36 | 1.49 | .1760 |  | **Happy** | **6** | 24.95 | 4.16 | 21.84 | <.0001 |  |
| **Well-being** | 3 | 1.56 | .52 | 2.15 | .0919 |  | **Well-being** | **3** | 45.22 | 15.07 | 79.16 | <.0001 |  |
| **Satisfied w/life** | 2 | .13 | .06 | .27 | .7667 |  | **Satisfied w/life** | **2** | 3.21 | 1.61 | 8.43 | .0002 |  |
| **Quality of Life** | 2 | .19 | .09 | .39 | .6796 |  | **Quality of Life** | **2** | 17.89 | 8.95 | 46.97 | <.0001 |  |
| **Social Support** | 2 | 1.18 | .59 | 2.43 | .0880 |  | **Social Support** | **2** | 697.02 | 348.51 | 1830.11 | <.0001 |  |
|  |  |  |  |  |  |  |  |  |  |  |  |  |  |
| **General Health** |  |  |  |  |  |  | **Likelihood of Depression** | |  |  |  |  |  |
| **Source** | **DF** | **Sum of Sqs** | **Mean Square** | **F Value*** | **Pr > F** |  | **Source** | **DF** | **Sum of Sqs** | **Mean Square** | **F Value*** | **Pr > F** |  |
| **Model** | 36 | 4054.35 | 112.62 | 530.88 | <.0001 |  | **Model** | 36 | 6177.89 | 171.61 | 736.01 | <.0001 |  |
| **Error** | 86755 | 18404.32 | 0.21 |  |  |  | **Error** | 86755 | 20227.75 | 0.23 |  |  |  |
| **Corrected Total** | 86791 | 22458.67 |  |  |  |  | **Corrected Total** | 86791 | 26405.6337 |  |  |  |  |
| **R-Square** | **Coeff Var** | **Root MSE** | **Mean** |  |  |  | **R-Square** | **Coeff Var** | **Root MSE** | **Mean** |  |  |  |
| 0.1805 | 29.3064 | 0.4606 | 1.5716 |  |  |  | 0.233961 | 31.93691 | 0.482866 | 1.511937 |  |  |  |
| **Source** | **DF** | **Type III SS** | **Mean Square** | **F Value**** | **Pr > F** |  | **Source** | **DF** | **Type III SS** | **Mean Square** | **F Value**** | **Pr > F** |  |
| **Income** | 7 | 5.34 | 0.76 | 3.60 | 0.0007 |  | **Income** | 7 | 1.29 | 0.18 | 0.79 | 0.5944 |  |
| **Education** | 2 | 0.00 | 0.00 | 0.01 | 0.9911 |  | **Education** | 2 | 0.61 | 0.30 | 1.30 | **0.2724** |  |
| **Clubs** | 3 | 3.69 | 1.23 | 5.80 | 0.0006 |  | **Clubs** | 3 | 72.03 | 24.01 | 102.97 | <.0001 |  |
| **Religion** | 3 | 244.41 | 81.47 | 384.03 | <.0001 |  | **Religion** | 3 | 18.46 | 6.15 | 26.39 | <.0001 |  |
| **Depressed** | 2 | 33.43 | 16.71 | 78.79 | <.0001 |  | **Depressed** | 2 | 36.79 | 18.40 | 78.90 | <.0001 |  |
| **Live alone** | 2 | 1.43 | 0.71 | 3.36 | 0.0347 |  | **Live alone** | 2 | 11.96 | 5.98 | 25.64 | <.0001 |  |
| **Job** | 2 | 1.14 | 0.57 | 2.68 | 0.0685 |  | **Job** | 2 | 0.27 | 0.14 | 0.58 | 0.5573 |  |
| **Happy** | 6 | 76.12 | 12.69 | 59.81 | <.0001 |  | **Happy** | 6 | 127.59 | 21.26 | 91.20 | <.0001 |  |
| **Well-Being** | 3 | 144.04 | 48.01 | 226.33 | <.0001 |  | **Well-Being** | 3 | 1299.63 | 433.21 | 1858.00 | <.0001 |  |
| **Satisfied w/life** | 2 | 68.07 | 34.03 | 160.42 | <.0001 |  | **Satisfied w/life** | 2 | 34.69 | 17.34 | 74.39 | <.0001 |  |
| **Quality of Life** | 2 | 184.60 | 92.30 | 435.08 | <.0001 |  | **Quality of Life** | 2 | 1.45 | 0.72 | 3.10 | 0.0449 |  |
| **Social Support** | 2 | 13.90 | 6.95 | 32.77 | <.0001 |  | **Social Support** | 2 | 38.71 | 19.35 | 83.01 | <.0001 |  |
|  |  |  |  |  |  |  |  |  |  |  |  |  |  |
| **Happy** |  |  |  |  |  |  | **Emotional Well-being** | |  |  |  |  |  |
| **Source** | **DF** | **Sum of Sqs** | **Mean Square** | **F Value*** | **Pr > F** |  | **Source** | **DF** | **Sum of Sqs** | **Mean Square** | **F Value*** | **Pr > F** |  |
| **Model** | 32 | 56703.72 | 1771.99 | 3313.54 | <.0001 |  | **Model** | 35 | 37004.09 | 1057.26 | 3425.56 | <.0001 |  |
| **Error** | 86759 | 46396.40 | 0.53 |  |  |  | **Error** | 86756 | 26776.24 | 0.31 |  |  |  |
| **Corrected Total** | 86791 | 103100.12 |  |  |  |  | **Corrected Total** | 86791 | 63780.33 |  |  |  |  |
| **R-Square** | **Coeff Var** | **Root MSE** | **Mean** |  |  |  | **R-Square** | **Coeff Var** | **Root MSE** | **Mean** |  |  |  |
| 0.550 | 16.20201 | 0.731282 | 4.513527 |  |  |  | 0.58018 | 24.79814 | 0.555552 | 2.240299 |  |  |  |
| **Source** | **DF** | **Type III SS** | **Mean Square** | **F Value**** | **Pr > F** |  | **Source** | **DF** | **Type III SS** | **Mean Square** | **F Value**** | **Pr > F** |  |
| **Income** | 7 | 1.86 | 0.27 | 0.50 | 0.8383 |  | **Income** | 7 | 2.20 | 0.31 | 1.02 | **0.4156** |  |
| **Education** | 2 | 0.07 | 0.04 | 0.07 | 0.9336 |  | **Education** | 2 | 0.90 | 0.45 | 1.46 | **0.2319** |  |
| **Clubs** | 3 | 62.69 | 20.90 | 39.08 | <.0001 |  | **Clubs** | 3 | 7.97 | 2.66 | 8.61 | <.0001 |  |
| **Religion** | 3 | 240.59 | 80.20 | 149.96 | <.0001 |  | **Religion** | 3 | 14.69 | 4.90 | 15.86 | <.0001 |  |
| **Health** | 2 | 160.15 | 80.07 | 149.74 | <.0001 |  | **Health** | 2 | 203.97 | 101.98 | 330.43 | <.0001 |  |
| **Depressed** | 2 | 163.67 | 81.83 | 153.03 | <.0001 |  | **Depressed** | 2 | 1759.20 | 879.60 | 2849.93 | <.0001 |  |
| **Live Alone** | 2 | 38.23 | 19.11 | 35.74 | <.0001 |  | **Live Alone** | 2 | 66.04 | 33.02 | 106.98 | <.0001 |  |
| **Job** | 2 | 0.39 | 0.19 | 0.36 | 0.6973 |  | **Job** | 2 | 0.36 | 0.18 | 0.59 | **0.5566** |  |
| **Well-Being** | 3 | 23864.58 | 7954.86 | 14875.20 | <.0001 |  | **Happy** | 6 | 15821.44 | 2636.91 | 8543.67 | <.0001 |  |
| **Satisfied w/life** | 2 | 320.58 | 160.29 | 299.73 | <.0001 |  | **Satisfied w/life** | 2 | 44.14 | 22.07 | 71.50 | <.0001 |  |
| **Quality of Life** | 2 | 128.88 | 64.44 | 120.5 | <.0001 |  | **Quality of Life** | 2 | 7.34 | 3.67 | 11.89 | <.0001 |  |
| **Social Support** | 2 | 402.86 | 201.43 | 376.66 | <.0001 |  | **Social Support** | 2 | 44.02 | 22.01 | 71.32 | <.0001 |  |
|  |  |  |  |  |  |  |  |  |  |  |  |  |  |
| **Satisfied with Life** |  |  |  |  |  |  | **Quality of Life** |  |  |  |  |  |  |
| **Source** | **DF** | **Sum of Sqs** | **Mean Square** | **F Value*** | **Pr > F** |  | **Source** | **DF** | **Sum of Sqs** | **Mean Square** | **F Value*** | **Pr > F** |  |
| **Model** | 36 | 14340.30 | 398.34 | 3989.36 | <.0001 |  | **Model** | 36 | 14044.73368 | 390.13149 | 3802.26 |  |  |
| **Error** | 86755 | 8662.56 | 0.10 |  |  |  | **Error** | 86755 | 8901.51035 | 0.10261 |  |  |  |
| **Corrected Total** | 86791 | 23002.86 |  |  |  |  | **Corrected Total** | 86791 | 22946.24403 |  |  |  |  |
| **R-Square** | **Coeff Var** | **Root MSE** | **Mean** |  |  |  | **R-Square** | **Coeff Var** | **Root MSE** | **Mean** |  |  |  |
| 0.623414 | 21.15093 | 0.315992 | 1.493986 |  |  |  | 0.612071 | 21.82784 | 0.32032 | 1.467485 |  |  |  |
| **Source** | **DF** | **Type III SS** | **Mean Square** | **F Value**** | **Pr > F** |  | **Source** | **DF** | **Type III SS** | **Mean Square** | **F Value**** | **Pr > F** |  |
| **Income** | 7 | 0.73 | 0.10 | 1.05 | 0.3937 |  | **Income** | 7 | 2.04 | 0.29 | 2.84 | 0.0058 |  |
| **Education** | 2 | 0.12 | 0.06 | 0.61 | 0.5428 |  | **Education** | 2 | 0.04 | 0.02 | 0.19 | 0.8264 |  |
| **Clubs** | 3 | 1.34 | 0.45 | 4.48 | 0.0038 |  | **Clubs** | 3 | 2.44 | 0.81 | 7.94 | <.0001 |  |
| **Religion** | 3 | 12.57 | 4.19 | 41.97 | <.0001 |  | **Religion** | 3 | 6.45 | 2.15 | 20.97 | <.0001 |  |
| **Health** | 2 | 58.42 | 29.21 | 292.56 | <.0001 |  | **Health** | 2 | 92.15 | 46.07 | 449.04 | <.0001 |  |
| **Depressed** | 2 | 17.81 | 8.91 | 89.20 | <.0001 |  | **Depressed** | 2 | 0.99 | 0.50 | 4.84 | 0.0079 |  |
| **Live Alone** | 2 | 1.70 | 0.85 | 8.49 | 0.0002 |  | **Live Alone** | 2 | 9.88 | 4.94 | 48.13 | <.0001 |  |
| **Job** | 2 | 0.28 | 0.14 | 1.39 | 0.2492 |  | **Job** | 2 | 0.09 | 0.04 | 0.42 | 0.6598 |  |
| **Happy** | 6 | 99.73 | 16.62 | 166.46 | <.0001 |  | **Happy** | 6 | 55.59 | 9.26 | 90.29 | <.0001 |  |
| **Well-being** | 3 | 18.00 | 6.00 | 60.09 | <.0001 |  | **Well-being** | 3 | 3.60 | 1.20 | 11.69 | <.0001 |  |
| **Quality of Life** | 2 | 6929.62 | 3464.81 | 34699.8 | <.0001 |  | **Satisfied w/life** | 2 | 7111.25 | 3555.62 | 34653.5 | <.0001 |  |
| **Social Support** | 2 | 73.76 | 36.88 | 369.36 | <.0001 |  | **Social Support** | 2 | 63.69 | 31.85 | 310.38 | <.0001 |  |
|  |  |  |  |  |  |  |  |  |  |  |  |  |  |
| **Social Support** |  |  |  |  |  |  |  |  |  |  |  |  |  |
| **Source** | **DF** | **Sum of Sqs** | **Mean Square** | **F Value*** | **Pr > F** |  |  |  |  |  |  |  |  |
| **Model** | 36 | 5433.58 | 150.93 | 629.69 | <.0001 |  |  |  |  |  |  |  |  |
| **Error** | 86755 | 20794.70 | 0.24 |  |  |  |  |  |  |  |  |  |  |
| **Corrected Total** | 86791 | 26228.28 |  |  |  |  |  |  |  |  |  |  |  |
| **R-Square** | **Coeff Var** | **Root MSE** | **Mean** |  |  |  |  |  |  |  |  |  |  |
| **Source** | **DF** | **Type III SS** | **Mean Square** | **F Value**** | **Pr > F** |  |  |  |  |  |  |  |  |
| **Income** | 7 | 1.45 | 0.21 | 0.86 | 0.5363 |  |  |  |  |  |  |  |  |
| **Education** | 2 | 0.56 | 0.28 | 1.17 | 0.3114 |  |  |  |  |  |  |  |  |
| **Clubs** | 3 | 2.64 | 0.88 | 3.67 | 0.0116 |  |  |  |  |  |  |  |  |
| **Religion** | 3 | 69.24 | 23.08 | 96.29 | <.0001 |  |  |  |  |  |  |  |  |
| **Health** | 2 | 18.34 | 9.17 | 38.25 | <.0001 |  |  |  |  |  |  |  |  |
| **Depressed** | 2 | 49.26 | 24.63 | 102.75 | <.0001 |  |  |  |  |  |  |  |  |
| **Live Alone** | 2 | 987.65 | 493.83 | 2060.23 | <.0001 |  |  |  |  |  |  |  |  |
| **Job** | 2 | 0.27 | 0.13 | 0.56 | 0.5706 |  |  |  |  |  |  |  |  |
| **Happy** | 6 | 241.58 | 40.26 | 167.97 | <.0001 |  |  |  |  |  |  |  |  |
| **Well-being** | 3 | 46.64 | 15.55 | 64.86 | <.0001 |  |  |  |  |  |  |  |  |
| **Satisfied w/life** | 2 | 143.20 | 71.60 | 298.72 | <.0001 |  |  |  |  |  |  |  |  |
| **Quality of Life** | 2 | 133.447 | 66.724 | 278.37 | <.0001 |  |  |  |  |  |  |  |  |

| \| ***F= Mean Square of the Dependent Variable/DF** \| \| \| \|  \|  \|  \|  \| \| --- \| --- \| --- \| --- \| --- \| --- \| --- \| --- \| \| ****F= Mean Square of the Independent Variable/Mean Square Error of the Dependent Variable.** \| \| \| \| \| \| \|  \| \| **Pr<F is P value** \| \|  \|  \|  \|  \|  \|  \| \| **Type III SS is the sum of squares for a balanced test of each effect, adjusted for every other effect.** \| \| \| \| \| \| \| \| \|  \|  \|  \|  \|  \|  \|  \|  \| | | | |  |  |  |  |
| --- | --- | --- | --- | --- | --- | --- | --- | --- | --- | --- | --- | --- | --- | --- | --- | --- | --- | --- | --- | --- | --- | --- | --- | --- | --- | --- | --- | --- | --- | --- | --- | --- | --- | --- | --- | --- | --- | --- | --- | --- | --- | --- | --- | --- | --- | --- | --- |
|  | | | | | | | |
|  | |  |  |  |  |  |  |
|  | | | | | | | |
|  |  |  |  |  |  |  |  |
